# Ethanol selectively disrupts neuronal microexon regulation and chromatin marks in PC12 cells

**DOI:** 10.3389/fncel.2026.1854045

**Published:** 2026-07-08

**Authors:** Montserrat Olivares-Costa, Pascal Jorratt, Camila Morales, Sebastián F. Estay, Adrian R. Krainer, María Estela Andrés, Paola A. Haeger

**Affiliations:** 1 Departamento Ciencias Biomédicas, Facultad de Medicina, Universidad Católica del Norte, Coquimbo, Chile; 2 Cold Spring Harbor Laboratory, Cold Spring Harbor, NY, United States; 3 Facultad de Ciencias Biológicas, Pontificia Universidad Católica de Chile, Santiago, Chile; 4 Millennium Nucleus of Neuroepigenetics and Plasticity (EpiNeuro), Santiago, Chile

**Keywords:** alternative splicing, epigenetic regulation, ethanol neurotoxicity, microexon, neuronal differentiation

## Abstract

**Introduction:**

Exposure to alcohol (ethanol) is the most common toxic insult during human development and is a teratogenic agent associated with abnormalities ranging from behavioral disorders to fetal alcohol syndrome. Ethanol disrupts gene regulatory programs crucial for neuronal identity, including alternative splicing. A distinctive feature of developing neuronal transcriptomes is the high prevalence of microexon inclusion, which consists of short exonic sequences ranging from 3 to 51 nucleotides. However, the impact of ethanol on microexon inclusion remains poorly understood.

**Methods:**

In this study, we investigated whether ethanol exposure disrupts neuron-specific alternative splicing programs during neuronal differentiation. To this end, PC12 cells were exposed to 50 mM ethanol throughout five days of NGF-induced differentiation, alongside undifferentiated control cells, to analyze mRNA and protein expression dynamics of key splicing regulators and neurodevelopmental targets.

**Results:**

Our data show that in undifferentiated cells, ethanol exposure resulted in a significant reduction in *Srrm4* expression, while concomitantly increasing *Srrm3* mRNA levels, consistent with reduced neuron-specific microexon inclusion of the intersectin 1 (*Itsn*1) mRNA. While undifferentiated PC12 cells primarily express the canonical variants of lysine-specific demethylase 1 (LSD1) and PHD finger protein 21A (PHF21A), differentiation triggers the inclusion of microexons 8a and 14, generating the neuron-specific isoforms neuronal LSD1 and PHF21A, respectively. Under differentiated conditions, ethanol exposure increased neurite length but did not affect the mRNA levels of the neuronal variants of *Lsd1*, *Phf21a,* or *Itsn1*. Notably, ethanol exposure significantly increased the levels of LSD1 protein and its associated histone substrate, H3K4me2, in differentiated PC12 cells.

**Discussion:**

Taken together, these findings suggest that ethanol exposure modulates post-transcriptional and chromatin-based mechanisms during neuronal commitment and differentiation. This selective disruption highlights distinct molecular pathways that may contribute to persistent neurodevelopmental alterations relevant to alcohol use disorder, providing new insights into the teratogenic mechanisms of ethanol on the developing nervous system.

## Highlights

- Ethanol disrupts neuron-specific microexon inclusion in PC12 cells.
- SRRM3 and SRRM4 splicing factors are altered by ethanol exposure.
- LSD1 protein levels and H3K4me2 levels are elevated by ethanol in PC12 cells.
- Ethanol modulates post-transcriptional and chromatin-based mechanisms.

## 1 Introduction

Prenatal exposure to ethanol is a leading cause of neurodevelopmental disorders collectively known as fetal alcohol spectrum disorders (FASDs), which are characterized by persistent cognitive, behavioral, and neurological impairments (Chokroborty-Hoque et al., 2014; Lange et al., 2017; Popova et al., 2017). At the cellular level, ethanol disrupts multiple developmental processes, including neuronal proliferation, differentiation, migration, and synaptic maturation (Abrahao et al., 2017; Chen and Kannan, 2023; Dannenhoffer et al., 2021; Oskera et al., 2026). Moreover, beyond its well-established cytotoxic effects, accumulating evidence indicates that ethanol impairs neurodevelopment by perturbing gene regulatory programs critical for neuronal identity and plasticity, including post-transcriptional mechanisms that regulate neuronal gene expression (Basavarajappa, 2023; Kleiber et al., 2013; Ungerer et al., 2013).

One key post-transcriptional mechanism particularly vulnerable to developmental and environmental perturbations is alternative splicing. By generating different combinations of exons from a single gene, alternative splicing expands transcriptomic and proteomic diversity and enables context-dependent regulation of protein function. This process is especially prominent in the nervous system, where it contributes to neuronal differentiation, synaptic function, and plasticity (Nazim, 2024; Raj and Blencowe, 2015; Vuong et al., 2016). Importantly, splicing patterns in neurons are highly dynamic and responsive to external cues, allowing rapid adaptation of gene expression to changes in the cellular environment (Hermey et al., 2017; Thalhammer et al., 2020).

Among alternative splicing events, the inclusion of microexons, short exonic sequences typically ranging from 3 to 51 nucleotides, represents a distinctive feature of neuronal transcriptomes (Mackensen and Irimia, 2025). These highly conserved elements are preferentially included during neuronal differentiation and serve to fine-tune protein–protein interaction domains and signaling networks. Genome-wide studies have revealed that thousands of genes expressed during brain development contain microexons, many of which encode proteins involved in synaptic organization, vesicular trafficking, and cytoskeletal dynamics. Consistent with their functional relevance, neuronal microexon inclusion increases during early neurogenesis, and disruption of their regulation has been implicated in neurodevelopmental disorders, including autism spectrum disorders, highlighting the vulnerability of this splicing program to pathological perturbations (Irimia et al., 2014; Parada et al., 2021). Supporting the cell type-specific functional relevance of microexon regulation, a 9-nt microexon in the synaptic vesicle gene *unc-13* is skipped in olfactory neurons but included in motor neurons, suggesting that differential microexon inclusion contributes to the fine-tuning of neuronal function across distinct neuronal subtypes (Choudhary et al., 2025).

The precise regulation of neuronal microexons relies on the coordinated expression and activity of specialized splicing factors. In particular, SRRM4 (also known as nSR100) and its paralog SRRM3 play central roles in promoting neuron-specific microexon inclusion (Irimia et al., 2014; Torres-Méndez et al., 2019). Both proteins share structural domains that facilitate the recognition of intronic splicing enhancers and the recruitment of the spliceosome to microexon-containing transcripts, thereby enabling robust inclusion of neuron-specific exons (Torres-Méndez et al., 2019). However, SRRM4 is more broadly expressed during early neurogenesis and is essential for the activation of a wide repertoire of neuronal microexons, whereas SRRM3 expression is largely restricted to more mature neurons and appears to fine-tune microexon inclusion in a subset of transcripts. Despite these differences in temporal expression and target specificity, SRRM3 and SRRM4 exhibit partial functional redundancy, and the loss of either factor can be partially compensated by the other (Nakano et al., 2019). In addition to these specialized factors, other splicing regulators, such as NOVA1 and PTBP1, contribute to broader neuronal splicing programs, modulating alternative exons beyond the microexon subset (Li et al., 2015; Rusconi et al., 2015). Perturbations in the expression or function of these factors can selectively impair microexon inclusion. Furthermore, previous studies have shown that ethanol exposure can modulate splicing factor expression and alter splicing outcomes in neuronal systems (Downing et al., 2012; Fuentes-Beals et al., 2023; Kawasawa et al., 2017), suggesting that ethanol may selectively disrupt microexon regulation by interfering with splicing regulatory networks.

Several neurodevelopmentally relevant genes subject to neuron-specific microexon regulation exemplify how subtle splicing changes can fine-tune protein function. For instance, intersectin 1 (ITSN1), a multifunctional scaffold protein involved in endocytosis, synaptic vesicle recycling, and actin cytoskeleton remodeling, contains a neuron-specific microexon that modifies its binding affinity for key interactors, including dynamin-1 (Dyn1) (Dergai et al., 2010; Tsyba et al., 2004, 2008), thereby modulating endocytic dynamics at synapses. Similarly, the inclusion of a neuronal microexon in lysine-specific demethylase 1 (LSD1/KDM1A), a chromatin-modifying enzyme essential for neuronal differentiation and maturation, redirects its substrate specificity and reshapes transcriptional programs during neurodevelopment (Longaretti et al., 2020; Rusconi et al., 2015, 2016, 2017; Zibetti et al., 2010). In the case of PHF21A, a chromatin-associated factor implicated in transcriptional repression and intellectual disability, neuron-specific microexon inclusion disrupts nucleosome binding, suggesting a direct effect on chromatin engagement (Nagai et al., 2024; Porter et al., 2025). Collectively, these examples illustrate how microexon-mediated alternative splicing constitutes a versatile regulatory mechanism that coordinates synaptic function and epigenetic control in neurons.

In this context, we investigated whether ethanol exposure disrupts neuron-specific alternative splicing programs during neuronal differentiation, with particular emphasis on microexon regulation and its functional consequences. Focusing on key splicing regulators and neurodevelopmentally relevant targets, including *Itsn1*, *Lsd1*, and *Phf21a*, we examined how ethanol affects microexon inclusion and the expression of distinct splice isoforms. Given the established role of LSD1 in chromatin remodeling and neuronal maturation, we further explored whether ethanol-induced splicing alterations are accompanied by changes in epigenetic marks associated with transcriptional regulation. Together, this approach aims to uncover a mechanistic link between ethanol exposure, dysregulation of alternative splicing, and epigenetic remodeling, providing insight into how post-transcriptional and chromatin-based mechanisms converge to shape long-lasting neurodevelopmental outcomes relevant to FASD.

## 2 Materials and methods

### 2.1 Cell culture

PC12 cells, derived from a rat adrenal pheochromocytoma (*Rattus norvegicus*), were kindly provided by Dr. María Estela Andrés and originally obtained from the American Type Culture Collection (ATCC, Manassas, VA, USA). Cells were maintained at 37 °C in a humidified incubator with 5% CO₂ and cultured in Dulbecco’s Modified Eagle’s Medium (DMEM) supplemented with 10% horse serum (Gibco/Thermo Fisher Scientific, Grand Island, NY, USA, #1605012), 5% fetal bovine serum (HyClone/Cytiva, Logan, UT, USA; HyClone–Cytiva, #SV30160.03), and penicillin–streptomycin (Thermo Fisher Scientific, Waltham, MA, USA, #15140122). PC12 cells were seeded in 6-well plates at a density of 200,000 cells per well for mRNA and protein expression analyses. For immunostaining and whole-cell electrophysiological recordings, cells were seeded in 24-well plates at a density of 10,000 cells per well onto glass coverslips (Marienfeld Superior, Lauda-Königshofen, Germany, #0111520). The following day, the seeding medium was replaced with a differentiation medium consisting of DMEM supplemented with 2% horse serum and penicillin–streptomycin. Neuronal differentiation was induced by treatment with 50 ng/mL nerve growth factor (NGF) (Alomone Labs, Jerusalem, Israel, #N-100) (Sáez et al., 2015); this time point was considered day 1 of treatment. Cells were maintained under differentiation conditions for a total of 7 days (Supplementary Figure 1A). Half of the differentiation medium was replaced every other day, and an equivalent amount of NGF was added to the fresh medium to maintain a constant final concentration throughout the differentiation period. Control cells were cultured under identical conditions in the absence of NGF (undifferentiated PC12 cells). For ethanol treatment, PC12 cells were exposed daily to 50 mM ethanol (Sigma-Aldrich/Merck, Darmstadt, Germany) from day 2 to day 7 of differentiation for immunostaining and mRNA and protein analyses, and until day 5 for electrophysiological recordings (Supplementary Figure 1A).

### 2.2 RT-qPCR and rqf-PCR

After treatment, the culture medium was removed, and cells were washed with phosphate-buffered saline (PBS) before homogenization in TRIzol™ reagent (Invitrogen, Carlsbad, CA, USA, #15596018) for total RNA isolation. For cDNA synthesis, 1 μg of total RNA was reverse-transcribed using ImProm-II™ Reverse Transcriptase (Promega, Madison, WI, USA) according to the manufacturer’s standard protocol, with Oligo(dT)18 primers (Thermo Fisher Scientific, Waltham, MA, USA). Quantitative real-time PCR (RT–qPCR) was performed using an Applied Biosystems StepOne™ Real-Time PCR System with Brilliant II SYBR Green QPCR Master Mix (Agilent Technologies, Santa Clara, CA, USA). Relative quantity fluorescent PCR (rqf-PCR) was carried out as previously described (Zibetti et al., 2010). In brief, a 5′ FAM-labeled forward primer and an unlabeled reverse primer were used to amplify alternative splicing events. Amplified products were resolved by capillary electrophoresis under denaturing conditions at the Sequencing and Omics Technologies Facility of the Pontificia Universidad Católica de Chile. The relative abundance of each splice isoform was quantified as relative fluorescence units (RFU) using the Open-Source Independent Review and Interpretation System (OSIRIS) software (version 2.16; NCBI). Primer sequences used for RT–qPCR and rqf-PCR are listed in Supplementary Table S1.

### 2.3 Western blot

After the treatment, the culture medium was removed and cells were washed with PBS before homogenization in 300 μL of lysis buffer [containing 20 mM MOPS/Tris (pH 7.0), 0.3 M sucrose, 2 mM EDTA, 2 mM EGTA, 1% NP-40, and 0.1% sodium dodecyl sulfate (SDS)], supplemented with a protease inhibitor cocktail (Thermo Fisher Scientific, Waltham, MA, USA, #78430). The lysates were centrifuged at 5,000 × *g* for 10 min at 4 °C. Protein concentration was determined using the Pierce™ BCA Protein Assay Kit (Thermo Fisher Scientific, Waltham, MA, USA, #23227). Equal amounts of protein (50 μg) were mixed with loading buffer, denatured at 95 °C for 10 min, resolved by 10% sodium dodecyl sulfate–polyacrylamide gel electrophoresis (SDS–PAGE), and transferred onto 0.45 μm PVDF membranes (Thermo Fisher Scientific, Waltham, MA, USA, #88518).

Membranes were blocked for 1 h at room temperature in TBS-T (10 mM Tris–HCl, pH 8.0, 150 mM NaCl, 0.2% Tween 20) containing 5% non-fat dry milk and then incubated overnight at 4 °C with the following primary antibodies: anti-β-actin (1:5000, Thermo Fisher Scientific, Waltham, MA, USA); anti-LSD1(1:2500, Cell Signaling Technology, Danvers, MA, USA, C69G12); anti-ITSN1 (Abcam, Cambridge, UK, ab231338); anti-H3K4me2 and anti-H3K9me2 (1:1000, Abcam, Cambridge, UK, AB7766 and AB1220). After washing three times with TBS-T for 10 min each, membranes were incubated with horseradish peroxidase (HRP)-conjugated secondary antibodies for 1 h at room temperature and washed as described above. Immunoreactive bands were detected by chemiluminescence using SuperSignal™ West Femto Maximum Sensitivity Substrate (Thermo Fisher Scientific, Waltham, MA, USA, #34096). Images were acquired using the C-DiGit™ and Odyssey M imaging systems (LI-COR, Lincoln, NE, USA). Band intensities were quantified using ImageJ software.

### 2.4 Immunofluorescence

After treatment, the cells were rinsed three times with PBS and fixed with 4% paraformaldehyde (PFA). The cells were then washed once with PBS and permeabilized with 0.2% Triton X-100 in PBS for 15 min. Non-specific binding was blocked by incubation in 2% bovine serum albumin (BSA) in PBS containing 0.05% Triton X-100 for 1 h at room temperature. The cells were subsequently incubated overnight at 4 °C with the following primary antibodies diluted in 1% BSA in PBS containing 0.05% Triton X-100: rabbit polyclonal anti-intersectin-1 (Abcam, Cambridge, UK, ab231338), anti-MAP2 (Invitrogen, Carlsbad, CA, USA, 13–1,500), anti-H3K4me2 and anti-H3K9me2 (Abcam, Cambridge, UK, AB7766 and AB1220), and anti-LSD1 (Cell Signaling Technology, Danvers, MA, USA, C69G12). Coverslips were washed as described above and incubated with the appropriate fluorophore-conjugated secondary antibodies (1:1,000) for 1 h at room temperature. After a final wash, nuclei were counterstained with DAPI (1:500), and coverslips were mounted using Fluoromount™ Aqueous Mounting Medium (Sigma-Aldrich/Merck, St. Louis, MO, USA, #F4680).

### 2.5 Quantitative confocal microscopy

The images were captured using a laser scanning confocal microscope (LSCM-800), controlled by software ZEN-2.1. The laser emissions were at 405-, 488-, and 555-nm wavelengths, and the confocal images were obtained with a size of 1,024 × 1,024 pixels. A 40 × oil objective (NA = 1.3) was employed. The resulting binary images were then subjected to automatic thresholding, after which the fluorescence intensity was calculated through the use of the Fill Holes, Watershed, and Analyze Particles functions. The neurite tree morphology studies involved the acquisition of z-stacks at a step size of 0.8 μm, followed by the analysis of the resulting binary images using the SNT v3.2.11 plugin (Arshadi et al., 2021) for ImageJ 1.54f. All images were analyzed by an experimenter blinded to treatment conditions.

### 2.6 Whole-cell recordings

Whole-cell voltage-clamp recordings were made from DIV4 undifferentiated and differentiated PC12 cells in control conditions and after 4 days of treatment with ethanol. In brief, cells grown on coverslips were transferred to a submersion-type recording chamber perfused at 1–2 mL/min with a standard external recording solution (28 ± 1 °C), containing (in mM) 140 NaCl, 5 KCl, 2.5 CaCl2, 1 MgCl2, 10 HEPES, 10 D-glucose, and adjusted to pH 7.4. The cells were visualized using infrared differential interference contrast (DIC) on a Nikon Eclipse FN1 microscope. Whole-cell patch-clamp recordings were performed using patch electrodes (5–6 MΩ) filled with a potassium-based intracellular solution (Hu et al., 2018) containing (in mM) 140 KCl, 5 NaCl, 1 CaCl2, 5 EGTA, 10 HEPES, and adjusted to pH 7.4. All recordings started ~2 min after break-in to allow cell dialysis and stabilization. To elicit voltage-dependent currents, cells were maintained at a holding potential of −60 mV, and voltage-dependent currents were activated by increasing depolarizing pulses from −90 to 50 mV (10 mV steps; 100 ms). Leak currents were subtracted offline using a P/4 protocol. Signals were recorded using a Multiclamp 700B amplifier (Molecular Devices, San Jose, CA, USA), acquired at 20 kHz, and low-pass filtered at 2.4 kHz. Series resistance (*R_s_*) and input resistance (*R_in_*) were continuously monitored throughout the recordings, and *R_s_* was left uncompensated. The liquid junction potential was not corrected. Cells with *R_s_* changes >20% during the experiment were excluded from the analysis. Data were acquired and analyzed using a custom-made routine written in Igor Pro 6.37 (WaveMetrics, Portland, OR, USA). All experiments were performed within an hour. Statistical analysis: Normality of the data sets was assessed using the Shapiro–Wilk test. For parametric data, group comparisons were performed using analysis of variance (ANOVA) followed by a *post-hoc* Tukey’s test. For non-parametric data, comparisons were made using Kruskal–Wallis ANOVA followed by a *post-hoc* Dunn’s test, using OriginPro 2018 (OriginLab, USA). All electrophysiological values are reported as mean ± SEM.

### 2.7 Statistical analysis

Data are presented as mean ± standard error of the mean (SEM). Data normality was assessed using the Shapiro–Wilk test. For normally distributed data, statistical significance was evaluated using Student’s *t*-test, whereas non-normally distributed data were analyzed using the Mann–Whitney *U* test. A significance threshold of *p* < 0.05 was applied. Additional statistical details are provided in the corresponding figure legends. All statistical analyses were performed using GraphPad Prism software (version 8.0.2).

## 3 Results

### 3.1 Ethanol selectively disrupts microexon regulation and splicing factor expression in undifferentiated PC12 cells

Alterations in alternative splicing have been proposed as a contributing mechanism in the pathogenesis of FASD (Arshadi et al., 2021; Donadoni et al., 2019; Fischer et al., 2021; Fuentes-Beals et al., 2023). To evaluate whether ethanol exposure affects the expression of splicing regulators involved in neurodevelopment, we subjected undifferentiated PC12 cells to continuous exposure to 50 mM ethanol for 5 days and quantified the mRNA levels of the neuronal splicing factors *Srrm4*, *Srrm3*, *Nova1*, and *Ptbp1*.

**Figure 1:**
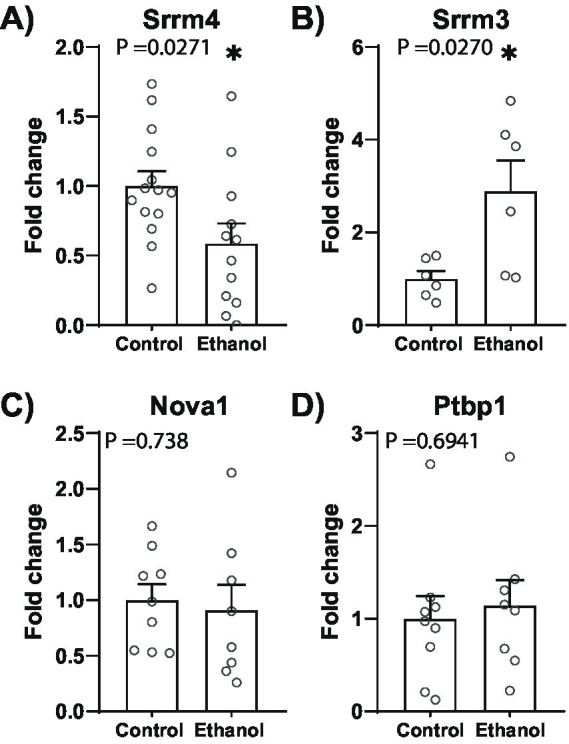
Ethanol selectively alters the expression of splicing factors in PC12 cells. Quantitative real-time PCR (RT-qPCR) was used to assess the mRNA expression levels of **(A)** Srrm4, **(B)** Srrm3, **(C)** Nova1, and **(D)** Ptbp1 in PC12 cells following continuous ethanol exposure. Data are represented as mean ± SEM from at least three independent experiments and analyzed using a t-test. ^*^p < 0.05.

Ethanol exposure resulted in a significant reduction in *Srrm4* expression, while concomitantly increasing *Srrm3* mRNA levels. In contrast, the expression of *Nova1* and *Ptbp1* remained unchanged (Figure 1). Overall, these findings indicate that ethanol causes a selective imbalance in the expression of *Srrm3* and *Srrm4* without affecting other major splicing factors such as *Nova1* and *Ptbp1*.

Given the established role of SRRM3 and SRRM4 in the regulation of microexon inclusion during neuronal differentiation (Torres-Méndez et al., 2019; Irimia et al., 2014), we next assessed the inclusion levels of three neuron-specific microexons: microexon *8a* in *Lsd1* transcripts, microexon *20* in *Itsn1*, and microexon *14* in *Phf21a*.

**Figure 2:**
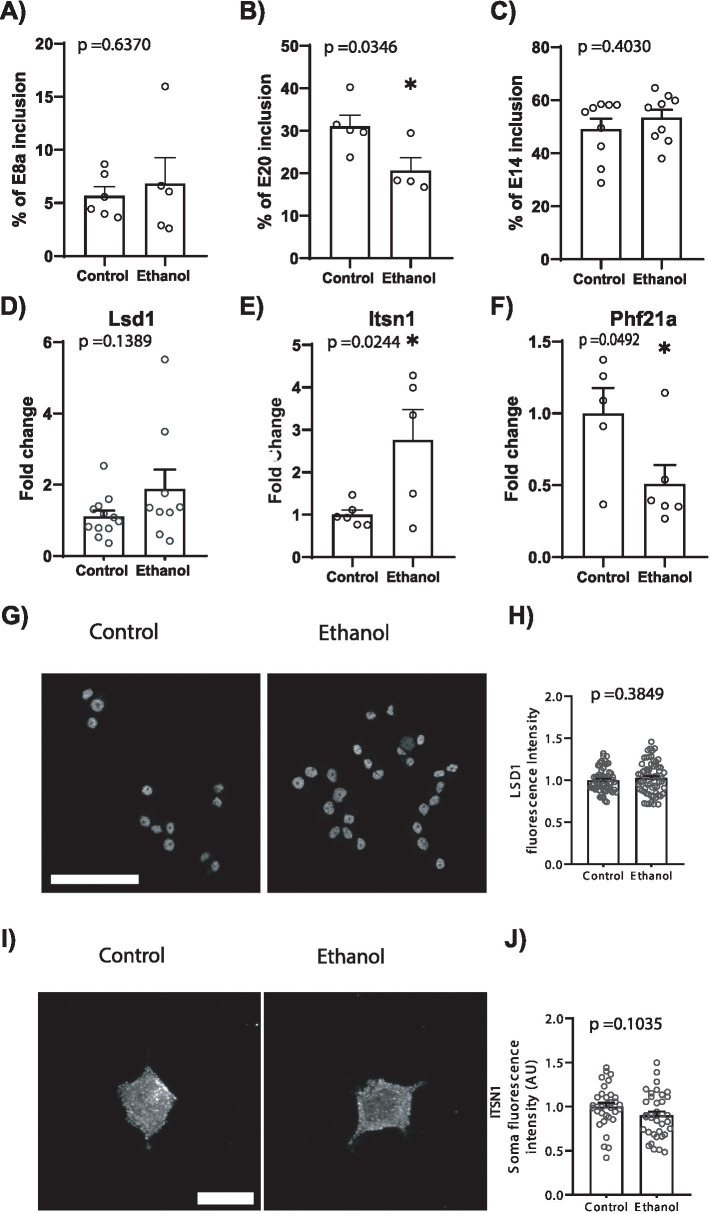
Inclusion of selected microexons in PC12 cells continuously exposed to ethanol. **(A)** Inclusion of microexon 8a (E8a) in the Lsd1 transcript measured by rqf-PCR. **(B)** Inclusion of microexon 20 (E20) in the Itsn1 transcript measured by rqf-PCR. **(C)** Inclusion of microexon 14 (E14) in the Phf21a transcript measured by RT-PCR and confirmed by gel electrophoresis. **(D)** Total Lsd1 transcript levels. **(E)** Total Itsn1 transcript levels. **(F)** Total Phf21a transcript levels measured by RT-qPCR. Data are presented as mean ± S.E.M. from at least three independent experiments and analyzed using a t-test; *p < 0.05. **(G)** Representative confocal photomicrographs of undifferentiated PC12 cells immunolabeled with an anti-LSD1 antibody under control conditions or following ethanol treatment. **(H)** Quantification of total LSD1 fluorescence intensity. Data are expressed as fold change relative to control conditions. Scale bar, 50 μm. n = 60–63 randomly selected fields per condition from three independent biological replicates. Each dot represents the mean fluorescence intensity of an individual field. **(I)** Representative confocal photomicrographs of undifferentiated PC12 cells immunolabeled with anti-ITSN1 antibody under control conditions or following ethanol treatment. **(J)** Quantification of total ITNS1 fluorescence intensity in the soma. Data are expressed as fold change relative to control conditions. Scale bar, 10 μm.33-36 randomly selected soma. Each dot represents the mean fluorescence intensity of an individual field.

Ethanol exposure did not alter the inclusion of microexon 8a in Lsd1 or microexon *14* in *Phf21a* transcripts (Figures 2A,C). By contrast, inclusion of microexon *E20* in *Itsn1* was significantly reduced, and total *Itsn1* mRNA levels were increased following ethanol treatment (Figures 2B,E). ITSN1 protein levels, assessed by immunofluorescence, were unchanged (Figures 2I,J). Total *Lsd1* expression, as determined by RT-qPCR, Western blot, and immunofluorescence, was unchanged (Figures 2D,G,H; Supplementary Figure 1). In contrast, total *Phf21a* mRNA levels were significantly decreased after ethanol exposure (Figure 2F).

Collectively, these results indicate that ethanol exposure alters both the expression of key microexon splicing regulators and the inclusion of specific neuron-associated microexon in PC12 cells. The coordinated downregulation of *Srrm4* and upregulation of *Srrm3* is associated with reduced inclusion of the *Itsn1* microexon *20*, supporting a targeted disruption of microexon regulation rather than a global alteration of alternative splicing. In addition, the apparent discrepancies between mRNA and protein levels for ITSN1 and LSD1 should be interpreted with caution, as the lack of isoform-specific antibodies limits the precise quantification of microexon-containing protein isoforms. Nevertheless, we cannot rule out the possibility that ethanol exposure also affects additional layers of post-transcriptional and/or post-translational regulation.

### 3.2 Ethanol enhances neurite outgrowth and H3K4me2 levels without altering microexon splicing in NGF-differentiated PC12 cells

**Figure 3:**
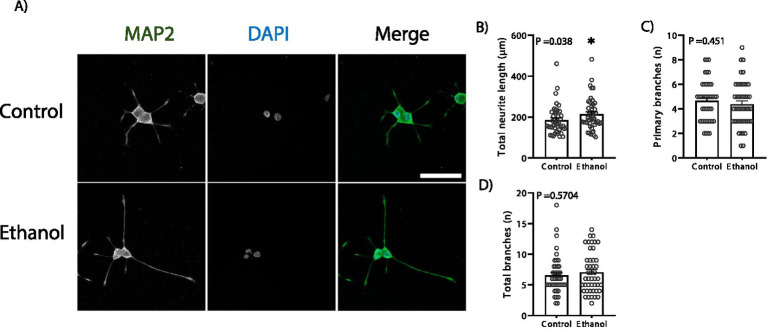
Ethanol increases neurite outgrowth in NGF-differentiated PC12 cells. **(A)** Representative confocal photomicrographs of NGF-differentiated PC12 cells immunolabeled with anti-MAP2 (green) and DAPI for nuclear staining (blue). **(B)** Quantification of total neurite length, **(C)** total number of primary branches, and **(D)** total branches in control or ethanol-treated NGF-differentiated PC12 cells. Scale bar, 50 μm. Data are represented as means ± SEM. *n* = 43–45 randomly chosen cells per treatment from three biological replicates. Only cells with a total neurite length greater than 100 μm were included in the analysis. ^*^p < 0.05, Mann–Whitney *U* test.

Given that microexon inclusion occurs primarily during neuronal differentiation, we next examined the effects of continuous ethanol exposure on cell morphology and gene expression after NGF-induced PC12 cell differentiation (Figure 3A). Morphological analysis revealed that ethanol-treated differentiated PC12 cells exhibited a significant increase in neurite length compared with control cells. In contrast, ethanol exposure did not significantly affect the number of primary neurites or the total number of neurite branches (Figure 3).

To further characterize the effects of ethanol in this differentiation model, we assessed the expression of synaptic structural plasticity-associated markers and examined electrophysiological properties in NGF-differentiated PC12 cells. The ethanol concentration used in these experiments (50 mM) corresponds to a relatively high level of exposure compared with typical human blood alcohol concentrations, approximating severe intoxication. In addition, because ethanol was administered repeatedly over 5 days but likely evaporated between administrations (Adams et al., 2023), the exposure paradigm was repeated yet intermittent, thereby resembling a binge-drinking-like pattern, which is common in individuals with problematic alcohol use. Under these conditions, although *Bdnf* and *Gap43* expression increased following NGF-induced differentiation (data not shown), ethanol treatment did not significantly affect the mRNA levels of these markers relative to differentiated control cells (Supplementary Figure 2). Consistent with this, whole-cell voltage-clamp recordings across membrane potentials ranging from −90 to +50 mV showed no significant differences in current at +50 mV between control and ethanol-exposed NGF-differentiated PC12 cells after 4 h of treatment (Supplementary Figure 3).

NGF-induced neuronal differentiation of PC12 cells was accompanied by an increase in the inclusion of neuron-specific microexons (Supplementary Figure 4), validating the use of this system as suitable for investigating the regulation of neuron-specific microexon inclusion under ethanol exposure. Surprisingly, expression levels of the splicing regulators *Srrm4*, *Srrm3*, *Nova1*, and *Ptbp1* remained unchanged after NGF-induced differentiation (data not shown).

**Figure 4:**
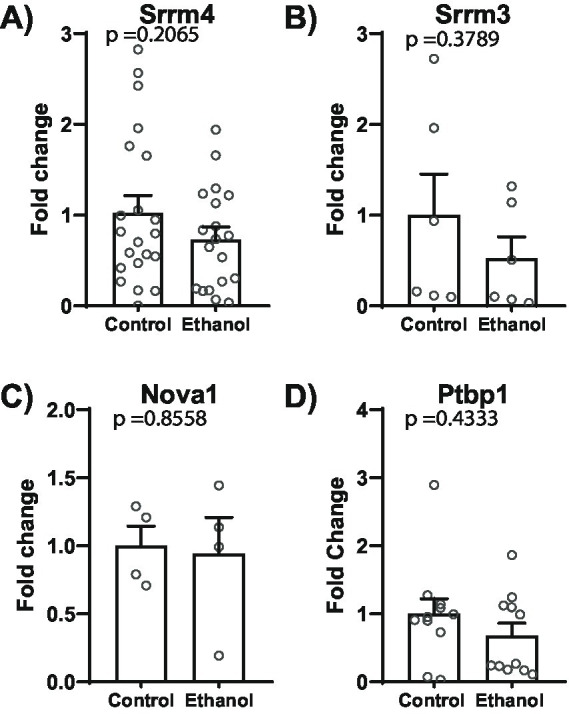
Ethanol selectively alters the expression of splicing factors in PC12 cells. Quantitative real-time PCR (RT-qPCR) was used to assess the mRNA expression levels of **(A)** *Srrm4,* **(B)** *Srrm3,* **(C)** *Nova1,* and **(D)** *Ptbp1* in PC12 cells following continuous ethanol exposure. Data are represented as mean ± SEM from at least three independent experiments and analyzed using a *t*-test. *p < 0.05.

Furthermore, ethanol exposure during the differentiation protocol did not produce significant changes in the expression of any of the splicing factors analyzed (Figure 4).

**Figure 5:**
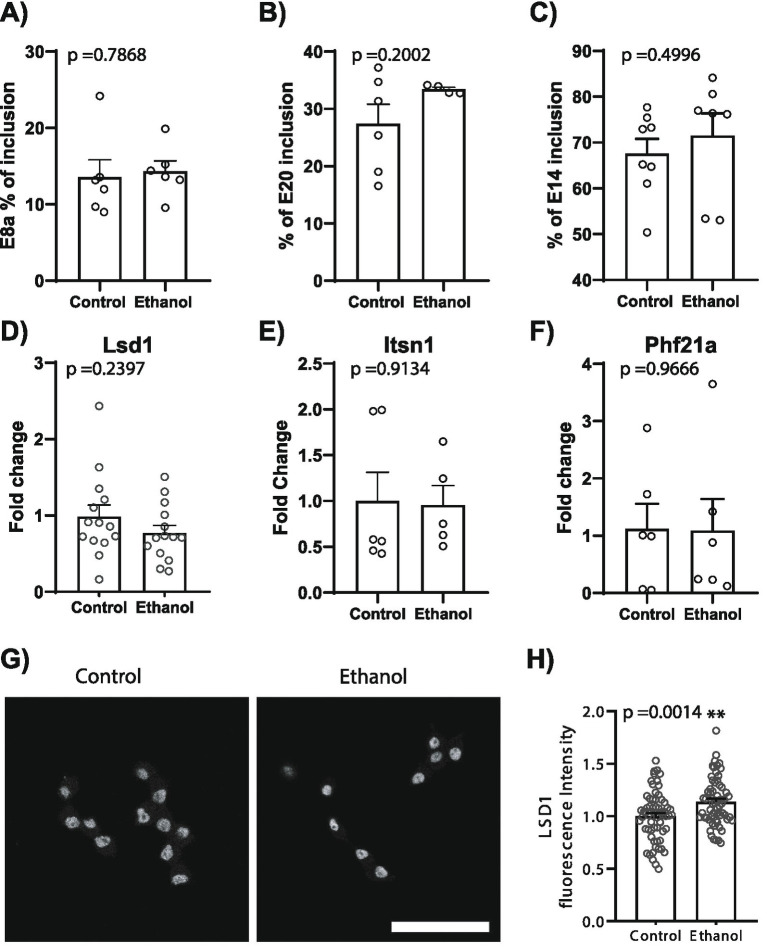
Ethanol does not alter neuron-specific microexon inclusion in NGF-differentiated PC12 cells. **(A)** Inclusion of microexon 8a in the *Lsd1* transcript measured by rqf-PCR. **(B)** Inclusion of microexon E20 in the *Itsn1* transcript measured by rqf-PCR. **(C)** Inclusion of microexon 14 in the *Phf21a* transcript measured by RT-PCR and confirmed by gel electrophoresis. **(D)** Total *Lsd1* transcript levels, **(E)** total *Itsn1* transcript levels, and **(F)** total *Phf21a* transcript levels measured by RT-qPCR. Data are presented as mean ± SEM from at least three independent experiments. **(G)** Representative confocal photomicrographs of NGF-differentiated PC12 cells immunostained with an anti-LSD1 antibody under control conditions or following ethanol treatment. **(H)** Quantification of total LSD1 fluorescence intensity in the soma. Data are expressed as fold change relative to control conditions. Scale bar, 50 μm. *n* = 60–63 randomly selected fields per condition from three independent biological replicates. Each dot represents the mean fluorescence intensity of an individual field.

We next examined whether ethanol affects neuron-specific alternative splicing in differentiated PC12 cells by analyzing the inclusion of microexons E8a in *Lsd1*, E20 in *Itsn1*, and E14 in *Phf21a*. No significant differences in microexon inclusion were observed between ethanol-treated and control differentiated cells (Figures 5A–C). Consistent with these findings, total *Itsn1* mRNA levels remained unchanged, and immunofluorescence analysis revealed preserved ITSN1 protein levels in both the soma and neurites (Figure 5E; Supplementary Figure 5). Similarly, *Phf21a* mRNA expression was not affected by ethanol exposure (Figure 5F). In contrast, immunofluorescence analysis revealed increased LSD1 protein levels following ethanol exposure (Figures 5G,H), whereas total *Lsd1* mRNA levels and total LSD1 protein levels assessed by Western blot remained unchanged (Figure 5D; Supplementary Figure 6).

Taken together, these data show differences in sensitivity to ethanol exposure between PC12 cells differentiated into neurons and undifferentiated PC12 cells with respect to alternative splicing.

**Figure 6:**
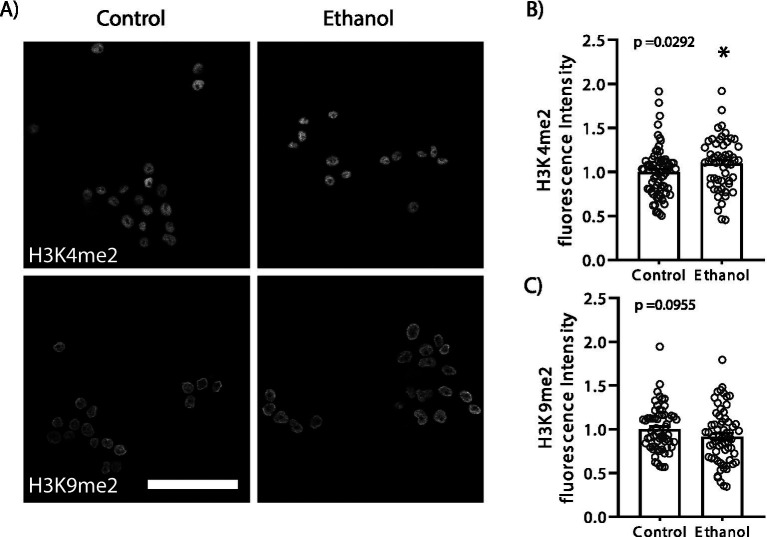
Fluorescence levels of histone H3 lysine 4 dimethylation (H3K4me2) and H3K9me2 in undifferentiated PC12 cells. **(A)** Representative confocal photomicrographs of undifferentiated PC12 cells under control conditions or following ethanol treatment, immunolabeled with antibodies against H3K4me2 and H3K9me2. **(B–C)** Quantification of H3K4me2 and H3K9me2 fluorescence intensity. Data are expressed as fold change relative to control conditions. Scale bar, 50 μm. Data are represented as means ± SEM. *n* = 53–68 randomly chosen fields per treatment from three biological replicates. Each dot represents the mean fluorescence intensity of an individual field. Kruskal–Wallis test followed by Dunn’s multiple-comparison test.

**Figure 7:**
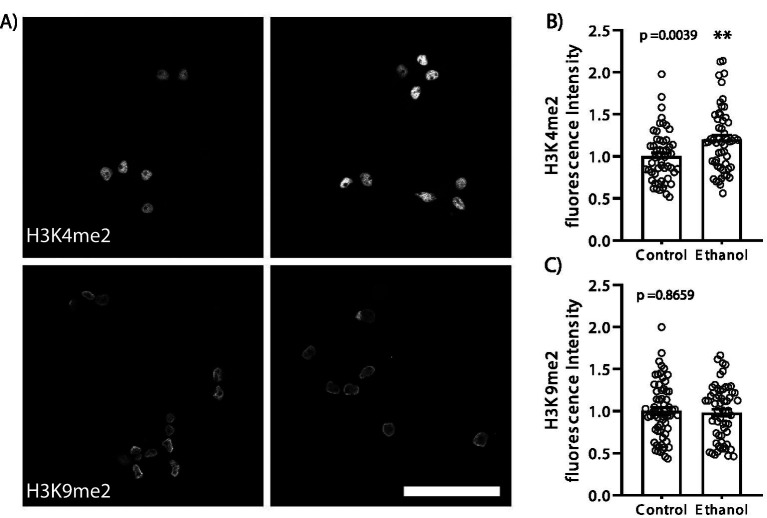
Fluorescence levels of histone H3 lysine 4 dimethylation (H3K4me2) and H3K9me2 in differentiated PC12 cells. **(A)** Representative confocal photomicrographs of NGF-differentiated PC12 cells under control conditions or following ethanol treatment, immunolabeled with antibodies against H3K4me2 and H3K9me2. Scale bar, 50 μm. **(B–C)** Quantification of H3K4me2 and H3K9me2 fluorescence intensity. Data are expressed as fold change relative to control conditions. Scale bar, 50 μm. Data are represented as means ± SEM. *n* = 53–68 randomly chosen fields per treatment from three biological replicates. Each dot represents the mean fluorescence intensity of an individual field. Kruskal–Wallis test followed by Dunn’s multiple-comparison test.

Considering the observed changes in LSD1 protein levels and given that LSD1 primarily demethylates lysine 4 and lysine 9 residues on histone H3 (H3K4 and H3K9), we next investigated whether ethanol exposure affects the methylation status of these histone marks. Immunofluorescence analysis showed that ethanol increased H3K4 dimethylation (H3K4me2) in both undifferentiated and NGF-differentiated PC12 cells (Figures 6A,B, 7A,B), whereas H3K9me2 levels were unchanged across all conditions (Figures 6A,C, 7A,C). Together, these results point to a selective effect of ethanol on chromatin regulation that is independent of the cellular differentiation state.

## 4 Discussion

In this study, we investigated how ethanol exposure affects neuron-specific alternative splicing programs and chromatin-associated regulatory mechanisms using PC12 cells as a model of neuronal differentiation. Our findings reveal that ethanol differentially impacts post-transcriptional and epigenetic regulation depending on the state of cellular differentiation.

In undifferentiated cells, ethanol altered the expression balance of key regulators of neuronal microexons, characterized by increased *Srrm3* and decreased *Srrm4* expression. The imbalance was associated with reduced inclusion of a neuron-specific microexon in *Itsn1* mRNA. Additionally, ethanol increased total *Itsn1* mRNA levels while decreasing total *Phf21a* mRNA levels. In contrast, during NGF-induced differentiation, ethanol did not significantly modify the expression of splicing factors or the inclusion of the microexons analyzed. However, ethanol consistently increased LSD1 protein levels, as determined by immunofluorescence, which revealed a predominantly nuclear localization that enabled reliable detection. Ethanol also increased H3K4me2 levels in both undifferentiated and differentiated cells. Together, these findings suggest that ethanol exerts stage-dependent effects, primarily by altering post-transcriptional and chromatin-associated regulatory mechanisms.

Neuronal microexons represent a specialized class of alternative splicing events that fine-tune protein function by modifying protein–protein interaction motifs. Despite their small size (3–51 nucleotides), their inclusion or exclusion can have significant functional consequences by altering interaction interfaces involved in neuronal signaling, cytoskeletal dynamics, and synaptic organization (Alam et al., 2026; Irimia et al., 2014; Roth et al., 2023; Ule and Blencowe, 2019). Genome-wide studies have demonstrated that neuronal microexons are tightly regulated during brain development and are particularly sensitive to perturbations in splicing regulatory networks (Irimia et al., 2014; Parada et al., 2021).

The splicing factors SRRM4 and SRRM3 are considered master regulators of microexon inclusion. SRRM4 is a well-established activator of neuronal microexon inclusion, whereas SRRM3 acts as a paralog that regulates overlapping but partially distinct subsets of exons (Lopez-Blanch et al., 2025; Torres-Méndez et al., 2019). In our study, ethanol exposure induced an imbalance in these regulators, with reduced *Srrm4* and increased *Srrm3* expression. This shift was associated with reduced inclusion of the *Itsn1* microexon, while microexons in *Lsd1* and *Phf21a* remained unchanged. The limited number of affected microexons is consistent with previous studies showing that individual microexons exhibit variable sensitivity to specific splicing factors and that partial redundancy between SRRM3 and SRRM4 can buffer splicing changes (Bonnal et al., 2025; Torres-Méndez et al., 2019). Therefore, ethanol-induced dysregulation may selectively affect a subset of neuronal microexons rather than globally disrupting microexon programs.

ITSN1 is a multidomain scaffold protein containing multiple SH3 domains that mediate interactions with proteins involved in membrane trafficking and endocytosis (Gubar et al., 2013; Kropyvko et al., 2010; Mintoo et al., 2024). The neuronal isoform, which includes a microexon adding five amino acids within an SH3 domain, has been proposed to enhance interactions with proteins involved in vesicle recycling (Dergai et al., 2010; Tsyba et al., 2004, 2008). Consistent with these observations, a recent study has shown that the neuronal *Itsn1* microexon contributes to neuritogenesis during zebrafish development (Lopez-Blanch et al., 2025). Although its role in PC12 cells remains unclear, studies in chromaffin cells, from which PC12 cells originate, have shown that pan-ITSN1 deletion impairs endocytic and vesicle trafficking processes (Yu et al., 2008), suggesting a similar functional role.

In our model, ethanol decreased inclusion of the neuronal ITSN1 microexon while increasing total *Itsn1* mRNA levels with no changes in ITSN1 protein levels. These findings suggest that ethanol may disrupt post-transcriptional regulatory processes controlling ITSN1 expression.

The disconnect between mRNA abundance and protein levels raises the possibility that microexon inclusion may influence protein stability or translation efficiency, although the precise mechanisms remain to be elucidated. Although the direct role of microexons in regulating protein stability or translational efficiency remains largely unknown, previous studies have suggested that microexon inclusion in specific transcripts can modulate translation-related pathways. For example, microexons have been described in genes such as *EIF4G* and *CPEB4*, both of which are involved in the regulation of mRNA translation and translational efficiency. In particular, EIF4G participates in the formation of the translation initiation complex through its interaction with eIF4E (Gonatopoulos-Pournatzis et al., 2020), while CPEB4 regulates poly(A) tail length and downstream mRNA translation (Garcia-Cabau et al., 2025). Therefore, although the precise mechanism underlying the altered ITSN1 expression observed here remains unclear, we cannot exclude the possibility that ethanol induces broader changes in the microexon inclusion patterns that indirectly affect translational regulation and/or protein stability of targets such as LSD1 or ITSN1.

### 4.1 Ethanol exposure modifies epigenetic processes

Beyond alternative splicing, our results indicate that ethanol also affects chromatin-associated regulatory pathways. LSD1 is a histone demethylase that targets H3K4me2 and H3K9me2 (Laurent et al., 2015; Wang et al., 2015; Zibetti et al., 2010); therefore, increased LSD1 expression would be expected to reduce H3K4me2 levels. Unexpectedly, ethanol exposure led to concurrent increases in both LSD1 protein and H3K4me2 levels in differentiated PC12 cells. This observation suggests that LSD1 abundance may not directly reflect its enzymatic activity or that additional chromatin regulatory mechanisms may dominate the balance of H3K4 methylation.

H3K4 methylation is dynamically regulated by the balance between demethylases such as LSD1 and methyltransferases of the MLL/SET family (Black et al., 2012; Hyun et al., 2017; Terzi Çizmecioğlu, 2024). Disruption of this balance may account for the increased H3K4me2 levels observed in ethanol-treated cells. Alternatively, the upregulation of LSD1 may represent a compensatory response to elevated H3K4me2, reflecting a feedback mechanism aimed at restoring chromatin homeostasis. Consistent with this interpretation, previous studies have reported that intermittent ethanol exposure increases *Lsd1* mRNA in the amygdala of adolescent rats (1 h after the last ethanol injection), without a corresponding increase in the neuronal isoform of *Lsd1* (Kyzar et al., 2017).

In addition, coordinated expression of neuronal splice variants of LSD1 and PHF21A has been proposed to impact the methylation landscape and transcriptional regulation during brain development. When neuronal microexons in both genes are included, the resulting complex displays reduced nucleosome binding and decreased nLSD1 enzymatic activity (Porter et al., 2025), potentially contributing to increased H3K4me2 levels. Although we did not detect ethanol-induced changes in the inclusion of these neuronal microexons in PC12 cells, alterations in LSD1 abundance may still influence chromatin dynamics and transcriptional regulation.

Our findings are also consistent with previous studies showing that ethanol promotes a more permissive chromatin state. ATAC-seq and RNA-seq analyses have revealed that acute ethanol exposure enhances chromatin accessibility in the amygdala (Krishnan et al., 2022). Similarly, acute ethanol exposure can decrease histone deacetylase (HDAC) activity while increasing histone acetyltransferase (HAT) CREB-binding protein (CBP), leading to increased acetylation of histones H3 and H4 in the amygdala (Pandey et al., 2008; Sakharkar et al., 2012). However, in contrast with some reports indicating ethanol-induced reductions in H3K9me2, we did not detect changes in this histone modification in PC12 cells, suggesting that ethanol-induced chromatin remodeling may vary depending on the cellular context and developmental stage.

### 4.2 Ethanol increases neurite branching

NGF-differentiated PC12 cells are widely described as sympathetic neuron-like catecholaminergic cells, predominantly exhibiting a noradrenergic phenotype (Chiba et al., 1981; Greene and Tischler, 1976).

In our study, ethanol exposure increased neurite length in NGF-differentiated PC12 cells. Previous studies have reported mixed effects of ethanol on neuronal morphology, including increased dendritic length and spine density in some brain regions (King et al., 1988; Klenowski et al., 2016) and reduced neuronal number and dendritic complexity in others (Clabough et al., 2021; Susick et al., 2014; Walker et al., 1981). Conversely, studies using PC12 cells have consistently shown that ethanol enhances NGF-induced neurite outgrowth. For instance, exposure to 100 mM ethanol promotes neurite formation (Messing et al., 1991) through PKC-dependent mechanisms (Roivainen et al., 1993) and by increasing microtubule polymerization and by increasing microtubule polymerization (Reiter-Funk and Dohrman, 2005). Indeed, it has been well established that under these conditions, such as 50 ng/mL NGF for 7 days, PC12 cells acquire a fully established neuronal differentiation (Schimmelpfeng et al., 2004). Importantly, our results demonstrate that a low ethanol concentration (50 mM), administered over 5 days, is sufficient to promote neurite outgrowth, suggesting that ethanol can modulate cytoskeletal remodeling even at moderate levels. The mechanism underlying ethanol-induced neurite branching remains incompletely understood. Interestingly, overexpression of the neuronal isoform of LSD1 increases neuronal branching in cultured cells, and nLSD1-null mice exhibit reduced synapse size (Toffolo et al., 2014; Zibetti et al., 2010), raising the possibility that increased LSD1 levels may contribute to the morphological changes observed. Additionally, coordinated regulation of neuronal splice variants has been implicated in structural transitions during neurogenesis (Dupas et al., 2026) and neuronal differentiation. In particular, neuronal PHF21A arises through developmentally regulated microexon splicing events (Nagai et al., 2024). Similarly, the differential neuronal inclusion/exclusion of a 9-nt microexon in *unc-13* mRNA is crucial for the proper function of motor and olfactory neurons (Choudhary et al., 2025). Together, these findings suggest that ethanol-induced alterations in chromatin regulation and alternative splicing may converge to influence neuronal differentiation and structural plasticity. Taken together, our findings reveal that ethanol exposure selectively interferes with neuronal microexon regulation while simultaneously altering chromatin-associated regulatory pathways. These effects are dependent on the differentiation state of the cells, with splicing alterations primarily observed in undifferentiated cells and chromatin changes occurring across both conditions. This dual impact on post-transcriptional and epigenetic regulation may represent an important mechanism by which ethanol disrupts neuronal development.

In the context of fetal alcohol spectrum disorders (FASD), PC12 cells provide a useful reductionist model to investigate the direct cellular effects of ethanol on neuronal differentiation, neurite outgrowth, apoptosis, and neurotrophic signaling (Oberdoerster and Rabin, 1999; Pantazis et al., 1992).

Although they do not fully recapitulate the complexity of developing neural tissue, they are widely considered a relevant system for mechanistic studies of ethanol-induced neurodevelopmental damage, particularly in catecholaminergic/sympathetic neuron-like cells (Chiba et al., 1981; Greene and Tischler, 1976).

In this context, despite the modest effects of ethanol on neuronal differentiation, our findings provide the first *in vitro* evidence that ethanol exposure differentially alters microexon inclusion in undifferentiated and differentiating PC12 cells, supporting the usefulness of this model for studying the impact of ethanol on alternative splicing programs.

Future studies should aim to determine the genome-wide impact of ethanol on neuronal microexon inclusion and chromatin modifications. Elucidating how ethanol perturbs the interplay between alternative splicing and epigenetic regulation during neuronal differentiation may provide new insights into the molecular basis of neurodevelopmental disorders associated with prenatal alcohol exposure, including fetal alcohol spectrum disorders.

## Data Availability

The raw data supporting the conclusions of this article will be made available by the authors, without undue reservation.

## Supplementary material

SUPPLEMENTARY FIGURE 1 LSD1 protein levels in undifferentiated PC12 cells under control and ethanol conditions. **(A)** Schematic representation of the experimental protocol for PC12 cell differentiation induced by NGF and subsequent ethanol treatment. **(B)** Total LSD1 protein levels assessed by Western blot. **(C)** Representative Western blot bands quantified in **(B)**. Data are represented as means ± S.E.M from five independent experiments.

SUPPLEMENTARY FIGURE 2 mRNA expression of synaptic structural plasticity-associated markers in PC12 cells. RT-qPCR was performed to assess the mRNA expression levels of **(A)** *Bdnf* and **(B)** *Gap43* in NGF-differentiated PC12 cells. Data are represented as means ± S.E.M from at least three independent experiments.

SUPPLEMENTARY FIGURE 3 Ethanol exposure in non-differentiated and differentiated PC12 cells does not alter electrophysiological properties. **(A)** Representative current traces evoked by depolarizing voltage steps in non-differentiated and differentiated (NGF-treated) PC12 cells, recorded in the absence or presence of ethanol. **(B)** Current–voltage (I–V) relationships constructed from steady-state current amplitudes measured at the end of each voltage step for all treatment groups. **(C)** Maximum current amplitude in response to a +50 mV step in all conditions; no significant differences were detected. **(D–F)** Series resistance, input resistance, and holding current, which reflect seal quality and membrane integrity, showed no differences between groups. Data are presented as mean ± SEM. Statistical analysis: one-way ANOVA followed by Tukey’s *post hoc* test; all comparisons were non-significant (*p* > 0.05).

SUPPLEMENTARY FIGURE 4 NGF treatment increases the inclusion of neuron-specific microexons in *Lsd1* and *Phf21a* transcripts in PC12 cells. **(A)** Inclusion of microexon 8a in the *Lsd1* transcript measured by rqf-PCR. **(B)** Inclusion of microexon E20 in the *Itsn1* transcript measured by rqf-PCR. **(C)** Inclusion of microexon 14 in the *Phf21a* transcript measured by RT-PCR and confirmed by gel electrophoresis. Data are represented as means ± S.E.M. ^*^*p*<0.05 by unpaired *t*-test.

SUPPLEMENTARY FIGURE 5 ITSN levels in differentiated PC12 cells under control and ethanol conditions. **(A)** Representative confocal photomicrographs of NGF-differentiated PC12 cells immunostained with an anti-ITSN1 antibody under control conditions or following ethanol treatment. **(B)** Quantification of total ITSN1 fluorescence intensity in the soma. Data are expressed as fold change relative to control conditions. **(C)** Quantification of the number of fluorescent puncta per 10 um area in neurites. **(D)** Quantification of the number of fluorescent puncta per area (μm^2^) in neurites **(E)**. Quantification of total ITSN1 fluorescence intensity in neurites. Data are expressed as fold change relative to control conditions. Scale bar, 50 μm. *n* = 60–63 randomly selected fields per condition from three independent biological replicates. Each dot represents the mean fluorescence intensity of an individual field.

SUPPLEMENTARY FIGURE 6 LSD1 levels in differentiated PC12 cells in the control and ethanol group. **(A)** Total LSD1 protein levels assessed by Western blot. **(B)** Representative Western blot bands quantified in **(A)**. Data are represented as means ± S.E.M from five independent experiments. ^**^*p* < 0.01 by unpaired *t*-test.

SUPPLEMENTARY Data sheet 1 Raw data of Western blots for LSD1.

## References

[ref1] Abrahao K. P. Salinas A. G. Lovinger D. M. (2017). Alcohol and the brain: neuronal molecular targets, synapses, and circuits. Neuron 96, 1223–1238. doi: 10.1016/j.neuron.2017.10.032, 29268093 PMC6566861

[ref2] Adams J. W. Negraes P. D. Truong J. Tran T. Szeto R. A. Guerra B. S. . (2023). Impact of alcohol exposure on neural development and network formation in human cortical organoids. Mol. Psychiatry 28, 1571–1584. doi: 10.1038/s41380-022-01862-7, 36385168 PMC10208963

[ref3] Alam S. Dermentzaki G. Cabrera-Garcia D. Li M. Wang R. Campbell M. . (2026). A neuron type-specific microexon in Ank3/ankyrin-G modulates calcium activity and neuronal excitability. Nat Commun. 17:3173. doi: 10.1038/s41467-026-69486-x, 41688438 PMC13046794

[ref4] Arshadi C. Günther U. Eddison M. Harrington K. I. S. Ferreira T. A. (2021). SNT: a unifying toolbox for quantification of neuronal anatomy. Nat. Methods 18, 374–377. doi: 10.1038/s41592-021-01105-7, 33795878

[ref5] Basavarajappa B. S. (2023). “Epigenetics in fetal alcohol spectrum disorder,” in Progress in Molecular Biology and Translational Science, eds. Singh V. Mani I. (Academic Press), 211–239. 10.1016/bs.pmbts.2023.01.004 37019593

[ref6] Black J. C. Van Rechem C. Whetstine J. R. (2012). Histone lysine methylation dynamics: establishment, regulation, and biological impact. Mol. Cell 48, 491–507. doi: 10.1016/j.molcel.2012.11.006, 23200123 PMC3861058

[ref7] Bonnal S. Bajew S. Martinez-Corral R. Irimia M. (2025). Core splicing architecture and early spliceosomal recognition determine microexon sensitivity to SRRM3/4. Nat. Struct. Mol. Biol. 32, 2022–2034. doi: 10.1038/s41594-025-01634-1, 40775527

[ref8] Chen S.-Y. Kannan M. (2023). Neural crest cells and fetal alcohol spectrum disorders: mechanisms and potential targets for prevention. Pharmacol. Res. 194:106855. doi: 10.1016/j.phrs.2023.106855, 37460002 PMC10528842

[ref9] Chiba T. Murata Y. Koike T. (1981). Plasticity of pheochromocytoma (PC12) cells demonstrated by nerve growth factor or glucocorticoid treatment: a catecholamine fluorescence and electron microscopic investigation. Biomed. Res. 2, 618–628. doi: 10.2220/biomedres.2.618

[ref10] Chokroborty-Hoque A. Alberry B. Singh S. M. (2014). Exploring the complexity of intellectual disability in fetal alcohol spectrum disorders. Front. Pediatr. 2:90. doi: 10.3389/fped.2014.00090, 25207264 PMC4143882

[ref11] Choudhary B. Napier-Jameson R. Norris A. (2025). Regulated microexon alternative splicing in single neurons tunes synaptic function. EMBO Rep. 26, 3640–3662. doi: 10.1038/s44319-025-00493-7, 40490601 PMC12287369

[ref12] Clabough E. Ingersoll J. Reekes T. Gleichsner A. Ryan A. (2021). Acute ethanol exposure during synaptogenesis rapidly alters medium spiny neuron morphology and synaptic protein expression in the dorsal striatum. Int. J. Mol. Sci. 23:290. doi: 10.3390/ijms23010290, 35008713 PMC8745582

[ref13] Dannenhoffer C. A. Robertson M. M. Macht V. A. Mooney S. M. Boettiger C. A. Robinson D. L. (2021). Chronic alcohol exposure during critical developmental periods differentially impacts persistence of deficits in cognitive flexibility and related circuitry. Int. Rev. Neurobiol. 160, 117–173. doi: 10.1016/bs.irn.2021.07.004, 34696872 PMC8674885

[ref14] Dergai M. Tsyba L. Dergai O. Zlatskii I. Skrypkina I. Kovalenko V. . (2010). Microexon-based regulation of ITSN1 and Src SH3 domains specificity relies on introduction of charged amino acids into the interaction interface. Biochem. Biophys. Res. Commun. 399, 307–312. doi: 10.1016/j.bbrc.2010.07.080, 20659428

[ref15] Donadoni M. Cicalese S. Sarkar D. K. Chang S. L. Sariyer I. K. (2019). Alcohol exposure alters pre-mRNA splicing of antiapoptotic mcl-1L isoform and induces apoptosis in neural progenitors and immature neurons. Cell Death Dis. 10:447. doi: 10.1038/s41419-019-1673-3, 31171771 PMC6554352

[ref16] Downing C. Flink S. Florez-McClure M. L. Johnson T. E. Tabakoff B. Kechris K. J. (2012). Gene expression changes in C57BL/6J and DBA/2J mice following prenatal alcohol exposure. Alcohol. Clin. Exp. Res. 36, 1519–1529. doi: 10.1111/j.1530-0277.2012.01757.x, 22530671 PMC3407322

[ref17] Dupas S. J. Parada G. E. Li J. D. Brown K. R. Moffat J. Blencowe B. J. (2026). Splice isoform-perturbation coupled to single cell transcriptome profiling reveals functions of microexons in neurogenesis and autism-linked pathways. Nat. Commun. 17:1217. doi: 10.1038/s41467-025-67931-x, 41633977 PMC12868881

[ref18] Fischer M. Chander P. Kang H. Mellios N. Weick J. P. (2021). Transcriptomic changes due to early, chronic intermittent alcohol exposure during forebrain development implicate WNT signaling, cell-type specification, and cortical regionalization as primary determinants of fetal alcohol syndrome. Alcohol. Clin. Exp. Res. 45, 979–995. doi: 10.1111/acer.14590, 33682149 PMC8643076

[ref19] Fuentes-Beals C. Olivares-Costa M. Andrés M. E. Haeger P. A. Riadi G. Oliva C. . (2023). Bioinformatic analysis predicts that ethanol exposure during early development causes alternative splicing alterations of genes involved in RNA post-transcriptional regulation. PLoS One 18:e0284357. doi: 10.1371/journal.pone.0284357, 37053190 PMC10101408

[ref20] Garcia-Cabau C. Bartomeu A. Tesei G. Cheung K. C. Pose-Utrilla J. Picó S. . (2025). Mis-splicing of a neuronal microexon promotes CPEB4 aggregation in ASD. Nature 637, 496–503. doi: 10.1038/s41586-024-08289-w, 39633052 PMC11711090

[ref21] Gonatopoulos-Pournatzis T. Niibori R. Salter E. W. Weatheritt R. J. Tsang B. Farhangmehr S. . (2020). Autism-Misregulated eIF4G microexons control synaptic translation and higher order cognitive functions. Mol. Cell 77, 1176–1192.e16. doi: 10.1016/j.molcel.2020.01.006, 31999954

[ref22] Greene L. A. Tischler A. S. (1976). Establishment of a noradrenergic clonal line of rat adrenal pheochromocytoma cells which respond to nerve growth factor. Proc. Natl. Acad. Sci. 73, 2424–2428. doi: 10.1073/pnas.73.7.2424, 1065897 PMC430592

[ref23] Gubar O. Morderer D. Tsyba L. Croisé P. Houy S. Ory S. . (2013). Intersectin: the crossroad between vesicle exocytosis and endocytosis. Front. Endocrinol. (Lausanne) 4:109. doi: 10.3389/fendo.2013.00109, 23986746 PMC3753573

[ref24] Hermey G. Blüthgen N. Kuhl D. (2017). Neuronal activity-regulated alternative mRNA splicing. Int. J. Biochem. Cell Biol. 91, 184–193. doi: 10.1016/j.biocel.2017.06.002, 28591617

[ref25] Hu R. Cao Q. Sun Z. Chen J. Zheng Q. Xiao F. (2018). A novel method of neural differentiation of PC12 cells by using Opti-MEM as a basic induction medium. Int. J. Mol. Med. 41, 195–201. doi: 10.3892/ijmm.2017.3195, 29115371 PMC5746309

[ref26] Hyun K. Jeon J. Park K. Kim J. (2017). Writing, erasing and reading histone lysine methylations. Exp. Mol. Med. 49:e324. doi: 10.1038/emm.2017.11, 28450737 PMC6130214

[ref27] Irimia M. Weatheritt R. J. Ellis J. D. Parikshak N. N. Gonatopoulos-Pournatzis T. Babor M. . (2014). A highly conserved program of neuronal microexons is misregulated in autistic brains. Cell 159, 1511–1523. doi: 10.1016/j.cell.2014.11.035, 25525873 PMC4390143

[ref28] Kawasawa Y. I. Mohammad S. Son A. I. Morizono H. Basha A. Salzberg A. C. . (2017). Genome-wide profiling of differentially spliced mRNAs in human fetal cortical tissue exposed to alcohol. Alcohol 62, 1–9. doi: 10.1016/j.alcohol.2017.05.001, 28755746 PMC7336896

[ref29] King M. A. Hunter B. E. Walker D. W. (1988). Alterations and recovery of dendritic spine density in rat hippocampus following long-term ethanol ingestion. Brain Res. 459, 381–385. doi: 10.1016/0006-8993(88)90656-7, 3179712

[ref30] Kleiber M. L. Mantha K. Stringer R. L. Singh S. M. (2013). Neurodevelopmental alcohol exposure elicits long-term changes to gene expression that alter distinct molecular pathways dependent on timing of exposure. J. Neurodev. Disord. 5:6. doi: 10.1186/1866-1955-5-6, 23497526 PMC3621102

[ref31] Klenowski P. M. Fogarty M. J. Shariff M. Belmer A. Bellingham M. C. Bartlett S. E. (2016). Increased synaptic excitation and abnormal dendritic structure of prefrontal cortex layer V pyramidal neurons following prolonged binge-like consumption of ethanol. eNeuro 3:ENEURO.0248-16.2016. doi: 10.1523/ENEURO.0248-16.2016, 28032119 PMC5179982

[ref32] Krishnan H. R. Zhang H. Chen Y. Bohnsack J. P. Shieh A. W. Kusumo H. . (2022). Unraveling the epigenomic and transcriptomic interplay during alcohol-induced anxiolysis. Mol. Psychiatry 27, 4624–4632. doi: 10.1038/s41380-022-01732-2, 36089615 PMC9734037

[ref33] Kropyvko S. Gerasymchuk D. Skrypkina I. Dergai M. Dergai O. Nikolaienko O. . (2010). Structural diversity and differential expression of novel human intersectin 1 isoforms. Mol. Biol. Rep. 37, 2789–2796. doi: 10.1007/s11033-009-9824-8, 19777371

[ref34] Kyzar E. J. Zhang H. Sakharkar A. J. Pandey S. C. (2017). Adolescent alcohol exposure alters lysine demethylase 1 (LSD1) expression and histone methylation in the amygdala during adulthood. Addict. Biol. 22, 1191–1204. doi: 10.1111/adb.12404, 27183824 PMC5110402

[ref35] Lange S. Rovet J. Rehm J. Popova S. (2017). Neurodevelopmental profile of fetal alcohol Spectrum disorder: a systematic review. BMC Psychol. 5:22. doi: 10.1186/s40359-017-0191-2, 28645298 PMC5481937

[ref36] Laurent B. Ruitu L. Murn J. Hempel K. Ferrao R. Xiang Y. . (2015). A specific LSD1/KDM1A isoform regulates neuronal differentiation through H3K9 demethylation. Mol. Cell 57, 957–970. doi: 10.1016/j.molcel.2015.01.010, 25684206 PMC4369399

[ref37] Li Y. I. Sanchez-Pulido L. Haerty W. Ponting C. P. (2015). RBFOX and PTBP1 proteins regulate the alternative splicing of micro-exons in human brain transcripts. Genome Res. 25, 1–13. doi: 10.1101/gr.181990.114, 25524026 PMC4317164

[ref38] Longaretti A. Forastieri C. Toffolo E. Caffino L. Locarno A. Misevičiūtė I. . (2020). LSD1 is an environmental stress-sensitive negative modulator of the glutamatergic synapse. Neurobiol. Stress 13:100280. doi: 10.1016/j.ynstr.2020.100280, 33457471 PMC7794663

[ref39] Lopez-Blanch L. Rodríguez-Marin C. Mantica F. Iñiguez L. P. Permanyer J. Kita E. M. . (2025). Phenotypic impact of individual conserved neuronal microexons and their master regulators in zebrafish. eLife 13:RP104275. doi: 10.7554/eLife.104275, 41252189 PMC12626424

[ref40] Mackensen T. Irimia M. (2025). From tiny exons to big insights: the expanding field of microexons. Annu. Rev. Genomics Hum. Genet. 26, 77–102. doi: 10.1146/annurev-genom-121323-103648, 40393474

[ref41] Messing R. O. Henteleff M. Park J. J. (1991). Ethanol enhances growth factor-induced neurite formation in PC12 cells. Brain Res. 565, 301–311. doi: 10.1016/0006-8993(91)91662-k, 1688193

[ref42] Mintoo M. Rajagopalan V. O’Bryan J. P. (2024). Intersectin—many facets of a scaffold protein. Biochem. Soc. Trans. 52, 1–13. doi: 10.1042/BST20211241, 38174740

[ref43] Nagai M. Porter R. S. Miyasato M. Wang A. Gavilan C. M. Hughes E. D. . (2024). Neuronal splicing of the unmethylated histone H3K4 reader, PHF21A, prevents excessive synaptogenesis. J. Biol. Chem. 300:107881. doi: 10.1016/j.jbc.2024.107881, 39395799 PMC11605454

[ref44] Nakano Y. Wiechert S. Bánfi B. (2019). Overlapping activities of two neuronal splicing factors switch the GABA effect from excitatory to inhibitory by regulating REST. Cell Rep. 27, 860–871.e8. doi: 10.1016/j.celrep.2019.03.072, 30995482 PMC6556397

[ref45] Nazim M. (2024). Post-transcriptional regulation of the transcriptional apparatus in neuronal development. Front. Mol. Neurosci. 17:1483901. doi: 10.3389/fnmol.2024.1483901, 39764514 PMC11701043

[ref46] Oberdoerster J. Rabin R. A. (1999). NGF-differentiated and undifferentiated PC12 cells vary in induction of apoptosis by ethanol. Life Sci. 64:PL267–PL272. doi: 10.1016/S0024-3205(99)00166-6, 10372659

[ref47] Oskera L. Charlet-Briart M. Tielens S. Nguyen L. Laguesse S. (2026). Impact of prenatal alcohol exposure on cerebral cortex development. Adv. Exp. Med. Biol. 1500, 143–181. doi: 10.1007/978-3-032-12741-9_6, 41478921

[ref48] Pandey S. C. Ugale R. Zhang H. Tang L. Prakash A. (2008). Brain chromatin remodeling: a novel mechanism of alcoholism. J. Neurosci. 28, 3729–3737. doi: 10.1523/JNEUROSCI.5731-07.2008, 18385331 PMC6671100

[ref49] Pantazis N. J. Dohrman D. P. Luo J. Goodlett C. R. West J. R. (1992). Alcohol reduces the number of pheochromocytoma (PC12) cells in culture. Alcohol 9, 171–180. doi: 10.1016/0741-8329(92)90048-F, 1605882

[ref50] Parada G. E. Munita R. Georgakopoulos-Soares I. Fernandes H. J. R. Kedlian V. R. Metzakopian E. . (2021). MicroExonator enables systematic discovery and quantification of microexons across mouse embryonic development. Genome Biol. 22:43. doi: 10.1186/s13059-020-02246-2, 33482885 PMC7821500

[ref51] Popova S. Lange S. Probst C. Gmel G. Rehm J. (2017). Estimation of national, regional, and global prevalence of alcohol use during pregnancy and fetal alcohol syndrome: a systematic review and meta-analysis. Lancet Glob. Health 5, e290–e299. doi: 10.1016/S2214-109X(17)30021-9, 28089487

[ref52] Porter R. S. An S. Gavilan M. C. Nagai M. Murata-Nakamura Y. Zhou B. . (2025). Coordinated neuron-specific splicing events restrict nucleosome engagement of the LSD1 histone demethylase complex. Cell Rep. 44:115213. doi: 10.1016/j.celrep.2024.115213, 39817906 PMC11864812

[ref53] Raj B. Blencowe B. J. (2015). Alternative splicing in the mammalian nervous system: recent insights into mechanisms and functional roles. Neuron 87, 14–27. doi: 10.1016/j.neuron.2015.05.004, 26139367

[ref54] Reiter-Funk C. K. Dohrman D. P. (2005). Chronic ethanol exposure increases microtubule content in PC12 cells. BMC Neurosci. 6:16. doi: 10.1186/1471-2202-6-16, 15762984 PMC555550

[ref55] Roivainen R. McMahon T. Messing R. O. (1993). Protein kinase C isozymes that mediate enhancement of neurite outgrowth by ethanol and phorbol esters in PC12 cells. Brain Res. 624, 85–93. doi: 10.1016/0006-8993(93)90063-s, 8252418

[ref56] Roth J. F. Braunschweig U. Wu M. Li J. D. Lin Z.-Y. Larsen B. . (2023). Systematic analysis of alternative exon-dependent interactome remodeling reveals multitasking functions of gene regulatory factors. Mol. Cell 83, 4222–4238.e10. doi: 10.1016/j.molcel.2023.10.034, 38065061

[ref57] Rusconi F. Grillo B. Ponzoni L. Bassani S. Toffolo E. Paganini L. . (2016). LSD1 modulates stress-evoked transcription of immediate early genes and emotional behavior. Proc. Natl. Acad. Sci. USA 113, 3651–3656. doi: 10.1073/pnas.1511974113, 26976584 PMC4822633

[ref58] Rusconi F. Grillo B. Toffolo E. Mattevi A. Battaglioli E. (2017). NeuroLSD1: splicing-generated epigenetic enhancer of neuroplasticity. Trends Neurosci. 40, 28–38. doi: 10.1016/j.tins.2016.11.002, 27986293

[ref59] Rusconi F. Paganini L. Braida D. Ponzoni L. Toffolo E. Maroli A. . (2015). LSD1 Neurospecific alternative splicing controls neuronal excitability in mouse models of epilepsy. Cereb. Cortex 25, 2729–2740. doi: 10.1093/cercor/bhu070, 24735673

[ref60] Sáez J. E. Gómez A. V. Barrios Á. P. Parada G. E. Galdames L. González M. . (2015). Decreased expression of CoREST1 and CoREST2 together with LSD1 and HDAC1/2 during neuronal differentiation. PLoS One 10:e0131760. doi: 10.1371/journal.pone.0131760, 26111147 PMC4482511

[ref61] Sakharkar A. J. Zhang H. Tang L. Shi G. Pandey S. C. (2012). Histone deacetylases (HDAC)-induced histone modifications in the amygdala: a role in rapid tolerance to the anxiolytic effects of ethanol. Alcohol. Clin. Exp. Res. 36, 61–71. doi: 10.1111/j.1530-0277.2011.01581.x, 21790673 PMC3208078

[ref62] Schimmelpfeng J. Weibezahn K.-F. Dertinger H. (2004). Quantification of NGF-dependent neuronal differentiation of PC-12 cells by means of neurofilament-L mRNA expression and neuronal outgrowth. J. Neurosci. Methods 139, 299–306. doi: 10.1016/j.jneumeth.2004.05.010, 15488244

[ref63] Susick L. L. Lowing J. L. Provenzano A. M. Hildebrandt C. C. Conti A. C. (2014). Postnatal ethanol exposure simplifies the dendritic morphology of medium spiny neurons independently of adenylyl cyclase 1 and 8 activity in mice. Alcohol. Clin. Exp. Res. 38, 1339–1346. doi: 10.1111/acer.12383, 24655226 PMC4106952

[ref64] Terzi Çizmecioğlu N. (2024). Roles and regulation of H3K4 methylation during mammalian early embryogenesis and embryonic stem cell differentiation. Adv. Exp. Med. Biol. 1470, 73–96. doi: 10.1007/5584_2023_794, 38231346

[ref65] Thalhammer A. Jaudon F. Cingolani L. A. (2020). Emerging roles of activity-dependent alternative splicing in homeostatic plasticity. Front. Cell. Neurosci. 14:104. doi: 10.3389/fncel.2020.00104, 32477067 PMC7235277

[ref66] Toffolo E. Rusconi F. Paganini L. Tortorici M. Pilotto S. Heise C. . (2014). Phosphorylation of neuronal lysine-specific demethylase 1LSD1/KDM1A impairs transcriptional repression by regulating interaction with CoREST and histone deacetylases HDAC1/2. J. Neurochem. 128, 603–616. doi: 10.1111/jnc.12457, 24111946

[ref67] Torres-Méndez A. Bonnal S. Marquez Y. Roth J. Iglesias M. Permanyer J. . (2019). A novel protein domain in an ancestral splicing factor drove the evolution of neural microexons. Nat. Ecol. Evol. 3, 691–701. doi: 10.1038/s41559-019-0813-6, 30833759

[ref68] Tsyba L. Gryaznova T. Dergai O. Dergai M. Skrypkina I. Kropyvko S. . (2008). Alternative splicing affecting the SH3A domain controls the binding properties of intersectin 1 in neurons. Biochem. Biophys. Res. Commun. 372, 929–934. doi: 10.1016/j.bbrc.2008.05.156, 18539136

[ref69] Tsyba L. Skrypkina I. Rynditch A. Nikolaienko O. Ferenets G. Fortna A. . (2004). Alternative splicing of mammalian Intersectin 1: domain associations and tissue specificities. Genomics 84, 106–113. doi: 10.1016/j.ygeno.2004.02.005, 15203208

[ref70] Ule J. Blencowe B. J. (2019). Alternative splicing regulatory networks: functions, mechanisms, and evolution. Mol. Cell 76, 329–345. doi: 10.1016/j.molcel.2019.09.017, 31626751

[ref71] Ungerer M. Knezovich J. Ramsay M. (2013). In utero alcohol exposure, epigenetic changes, and their consequences. Alcohol Res. 35, 37–46. doi: 10.35946/arcr.v35.1.05, 24313163 PMC3860424

[ref72] Vuong C. K. Black D. L. Zheng S. (2016). The neurogenetics of alternative splicing. Nat. Rev. Neurosci. 17, 265–281. doi: 10.1038/nrn.2016.27, 27094079 PMC4861142

[ref73] Walker D. W. Hunter B. E. Abraham W. C. (1981). Neuroanatomical and functional deficits subsequent to chronic ethanol Administration in Animals. Alcohol. Clin. Exp. Res. 5, 267–282. doi: 10.1111/j.1530-0277.1981.tb04901.x, 7018310

[ref74] Wang J. Telese F. Tan Y. Li W. Jin C. He X. . (2015). LSD1n is an H4K20 demethylase regulating memory formation via transcriptional elongation control. Nat. Neurosci. 18, 1256–1264. doi: 10.1038/nn.4069, 26214369 PMC4625987

[ref75] Yu Y. Chu P.-Y. Bowser D. N. Keating D. J. Dubach D. Harper I. . (2008). Mice deficient for the chromosome 21 ortholog Itsn1 exhibit vesicle-trafficking abnormalities. Hum. Mol. Genet. 17, 3281–3290. doi: 10.1093/hmg/ddn224, 18676989

[ref76] Zibetti C. Adamo A. Binda C. Forneris F. Toffolo E. Verpelli C. . (2010). Alternative splicing of the histone demethylase LSD1/KDM1 contributes to the modulation of neurite morphogenesis in the mammalian nervous system. J. Neurosci. 30, 2521–2532. doi: 10.1523/JNEUROSCI.5500-09.2010, 20164337 PMC6634524

