# Supplementary figures and images for: Ethanol selectively disrupts neuronal microexon regulation and chromatin marks in PC12 cells
Source: Front Cell Neurosci. 2026 Jul 8;20:1854045. doi: 10.3389/fncel.2026.1854045 (PMC13388176; doi:10.3389/fncel.2026.1854045)

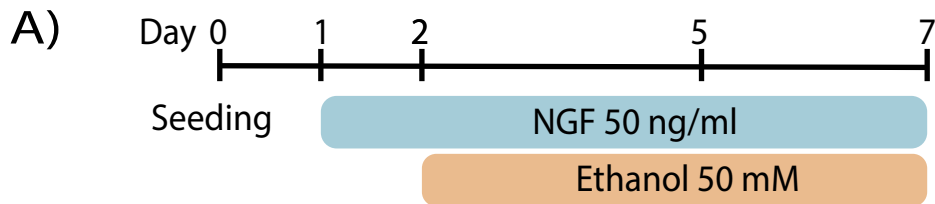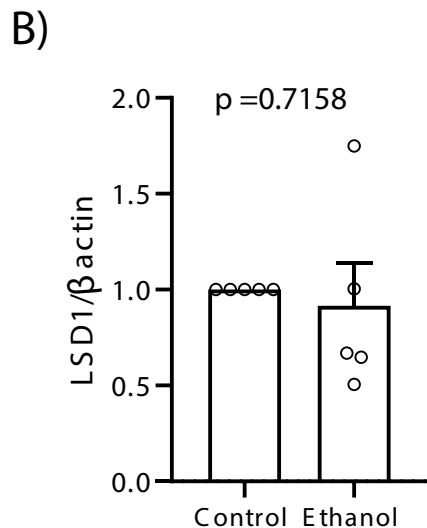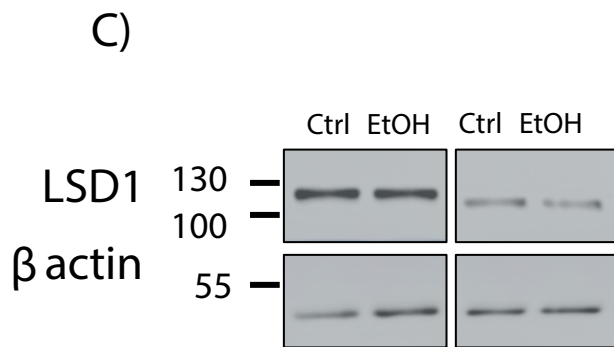

Supplement: SUPPLEMENTARY FIGURE 1 — LSD1 protein levels in undifferentiated PC12 cells under control and ethanol conditions. (A) Schematic representation of the experimental protocol for PC12 cell differentiation induced by NGF and subsequent ethanol treatment. (B) Total LSD1 protein levels assessed by Western blot. (C) Representative Western blot bands quantified in (B). Data are represented as means ± S.E.M from five independent experiments. [file Image_1.pdf]

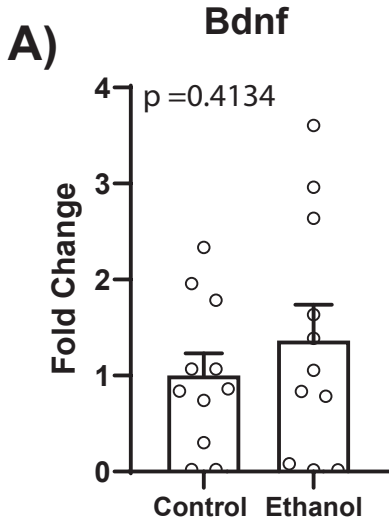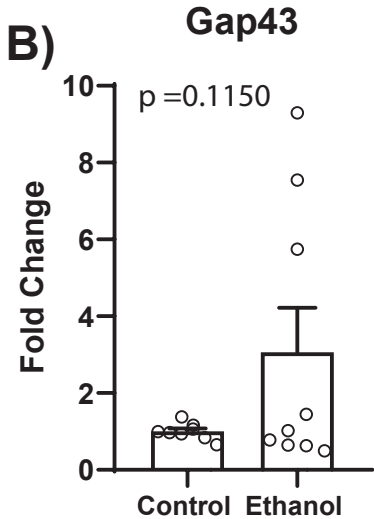

Supplement: SUPPLEMENTARY FIGURE 2 — mRNA expression of synaptic structural plasticity-associated markers in PC12 cells. RT-qPCR was performed to assess the mRNA expression levels of (A) Bdnf and (B) Gap43 in NGF-differentiated PC12 cells. Data are represented as means ± S.E.M from at least three independent experiments. [file Image_2.pdf]

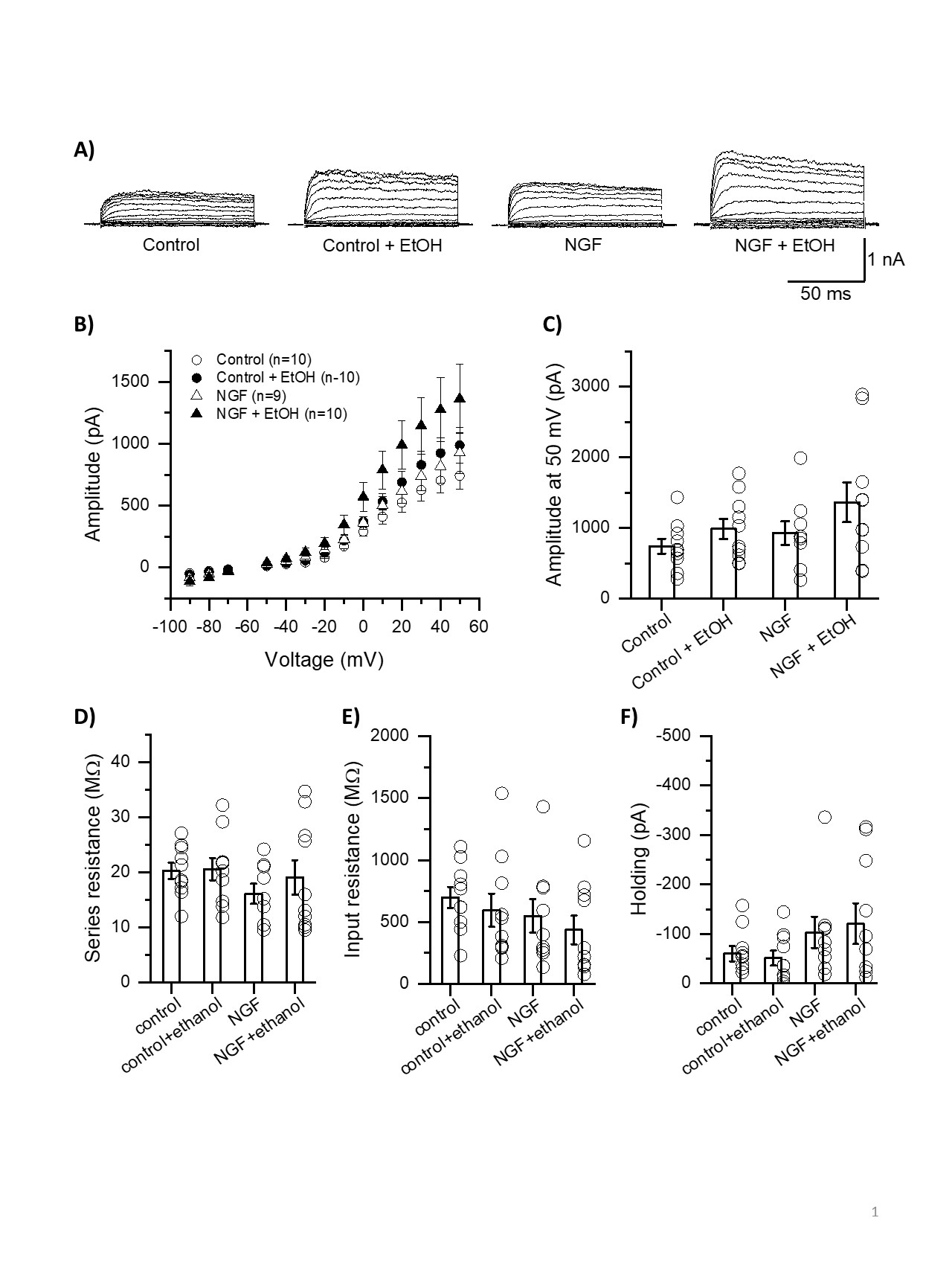

Supplement: SUPPLEMENTARY FIGURE 3 — Ethanol exposure in non-differentiated and differentiated PC12 cells does not alter electrophysiological properties. (A) Representative current traces evoked by depolarizing voltage steps in non-differentiated and differentiated (NGF-treated) PC12 cells, recorded in the absence or presence of ethanol. (B) Current–voltage (I–V) relationships constructed from steady-state current amplitudes measured at the end of each voltage step for all treatment groups. (C) Maximum current amplitude in response to a +50 mV step in all conditions; no significant differences were detected. (D–F) Series resistance, input resistance, and holding current, which reflect seal quality and membrane integrity, showed no differences between groups. Data are presented as mean ± SEM. Statistical analysis: one-way ANOVA followed by Tukey’s post hoc test; all comparisons were non-significant (p > 0.05). [file Image_3.JPEG]

**A)**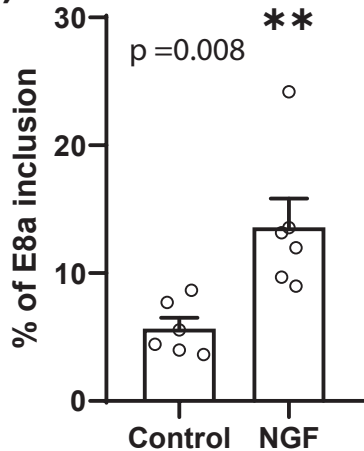**B)**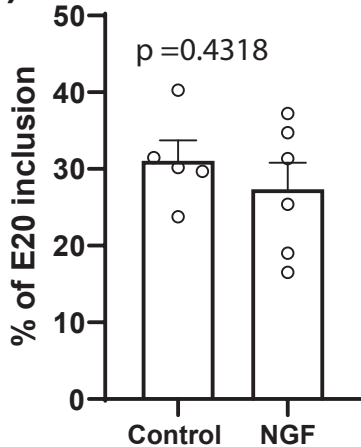**C)**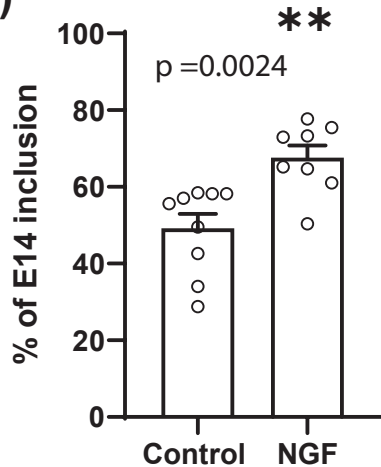

Supplement: SUPPLEMENTARY FIGURE 4 — NGF treatment increases the inclusion of neuron-specific microexons in Lsd1 and Phf21a transcripts in PC12 cells. (A) Inclusion of microexon 8a in the Lsd1 transcript measured by rqf-PCR. (B) Inclusion of microexon E20 in the Itsn1 transcript measured by rqf-PCR. (C) Inclusion of microexon 14 in the Phf21a transcript measured by RT-PCR and confirmed by gel electrophoresis. Data are represented as means ± S.E.M. *p<0.05 by unpaired t-test. [file Image_4.pdf]

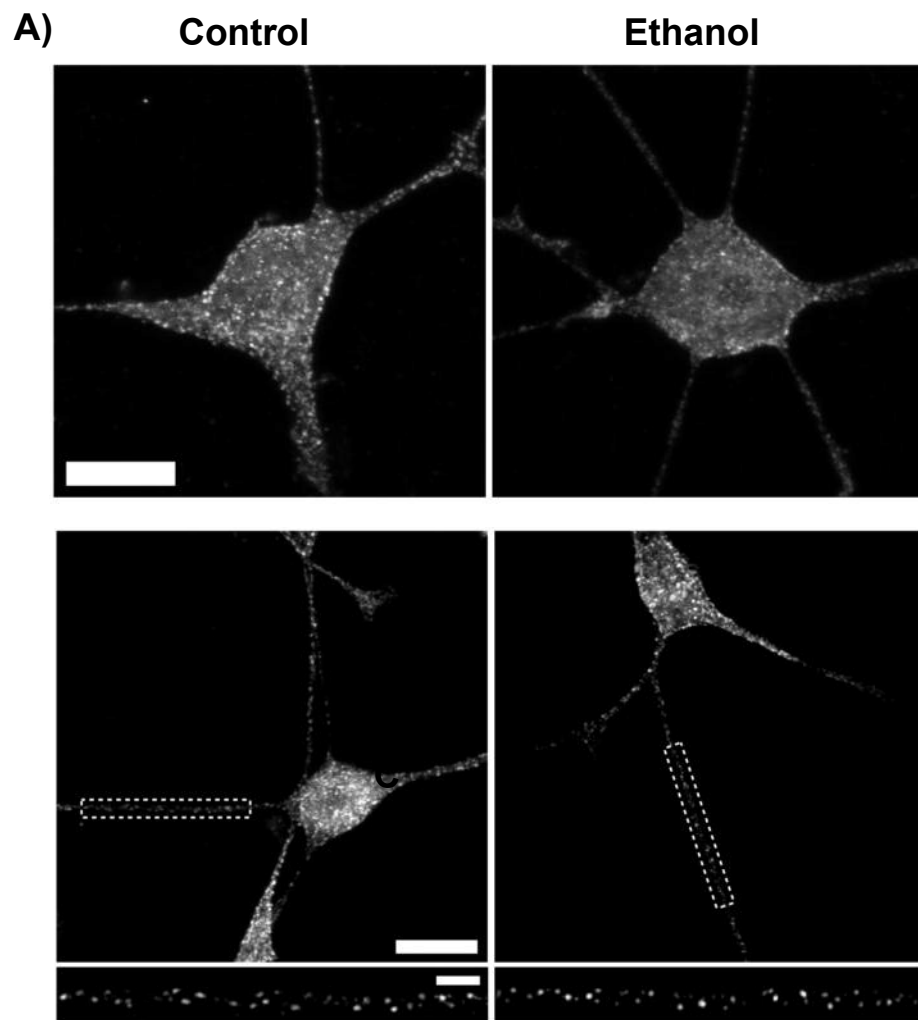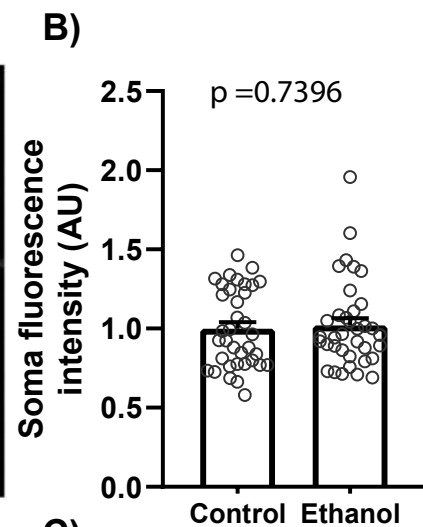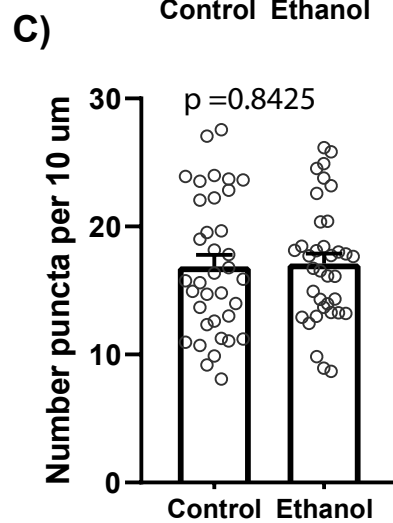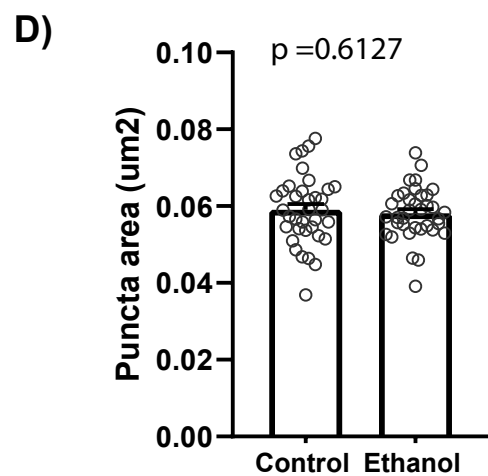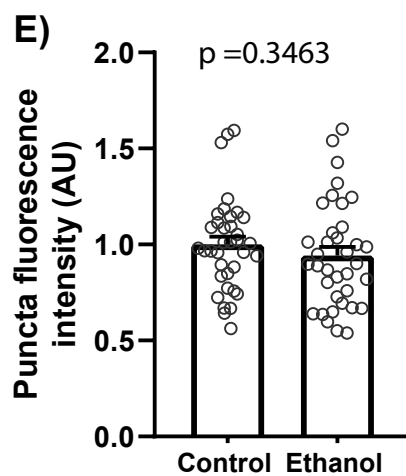

Supplement: SUPPLEMENTARY FIGURE 5 — ITSN levels in differentiated PC12 cells under control and ethanol conditions. (A) Representative confocal photomicrographs of NGF-differentiated PC12 cells immunostained with an anti-ITSN1 antibody under control conditions or following ethanol treatment. (B) Quantification of total ITSN1 fluorescence intensity in the soma. Data are expressed as fold change relative to control conditions. (C) Quantification of the number of fluorescent puncta per 10 um area in neurites. (D) Quantification of the number of fluorescent puncta per area (μm2) in neurites (E). Quantification of total ITSN1 fluorescence intensity in neurites. Data are expressed as fold change relative to control conditions. Scale bar, 50 μm. n = 60–63 randomly selected fields per condition from three independent biological replicates. Each dot represents the mean fluorescence intensity of an individual field. [file Image_5.pdf]

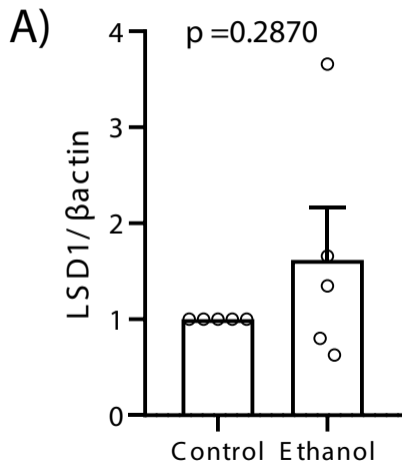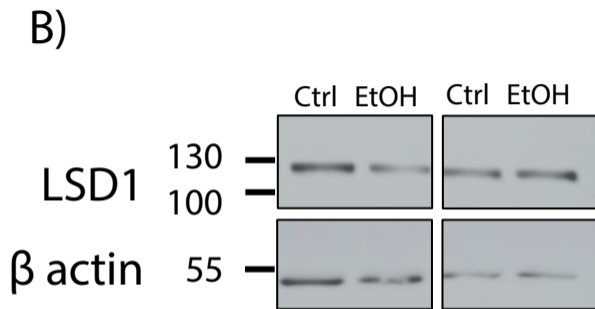

Supplement: SUPPLEMENTARY FIGURE 6 — LSD1 levels in differentiated PC12 cells in the control and ethanol group. (A) Total LSD1 protein levels assessed by Western blot. (B) Representative Western blot bands quantified in (A). Data are represented as means ± S.E.M from five independent experiments. **p < 0.01 by unpaired t-test. [file Image_6.pdf]
