# Supplementary material for: Ethanol selectively disrupts neuronal microexon regulation and chromatin marks in PC12 cells
Source: Front Cell Neurosci. 2026 Jul 8;20:1854045. doi: 10.3389/fncel.2026.1854045 (PMC13388176; doi:10.3389/fncel.2026.1854045)
Supplement: SUPPLEMENTARY Data sheet 1 — Raw data of Western blots for LSD1. [file Data_Sheet_1.PDF]

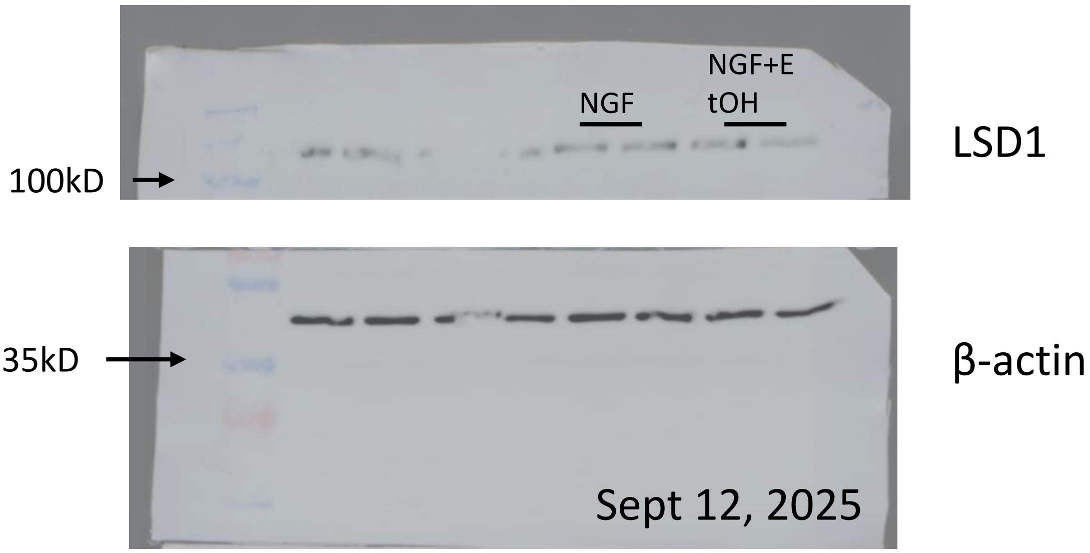
NGF
NGF+EtOH
LSD1
100kD
β-actin
35kD
Sept 12, 2025

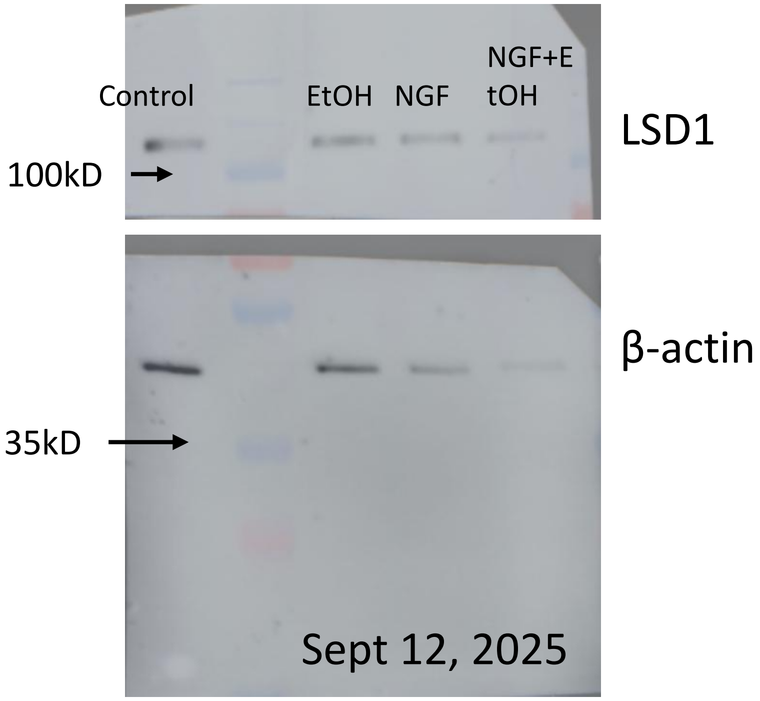
Control
EtOH
NGF
NGF+EtOH
LSD1
100kD
β-actin
35kD
Sept 12, 2025

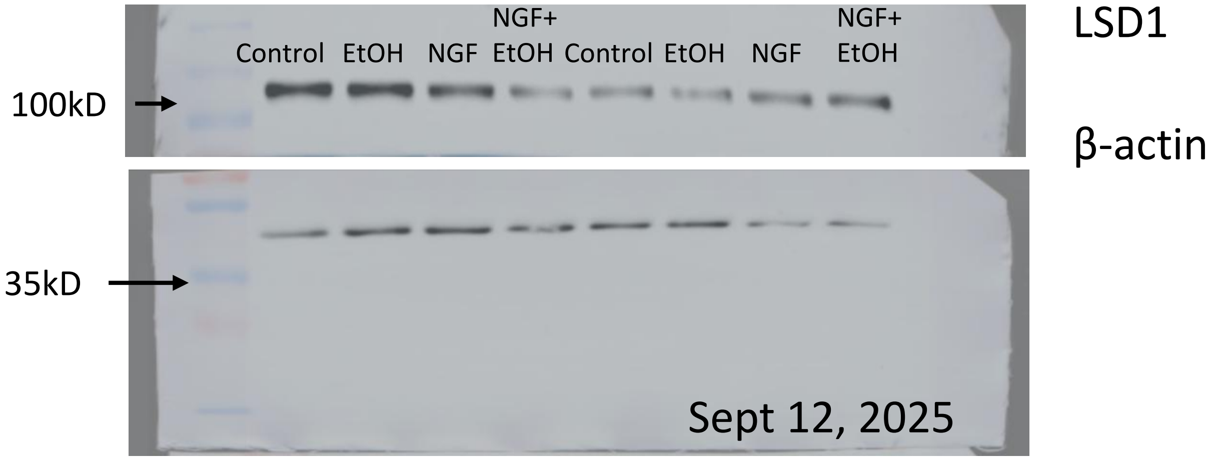
Control
EtOH
NGF
NGF+EtOH
Control
EtOH
NGF
NGF+EtOH
LSD1
100kD
β-actin
35kD
Sept 12, 2025

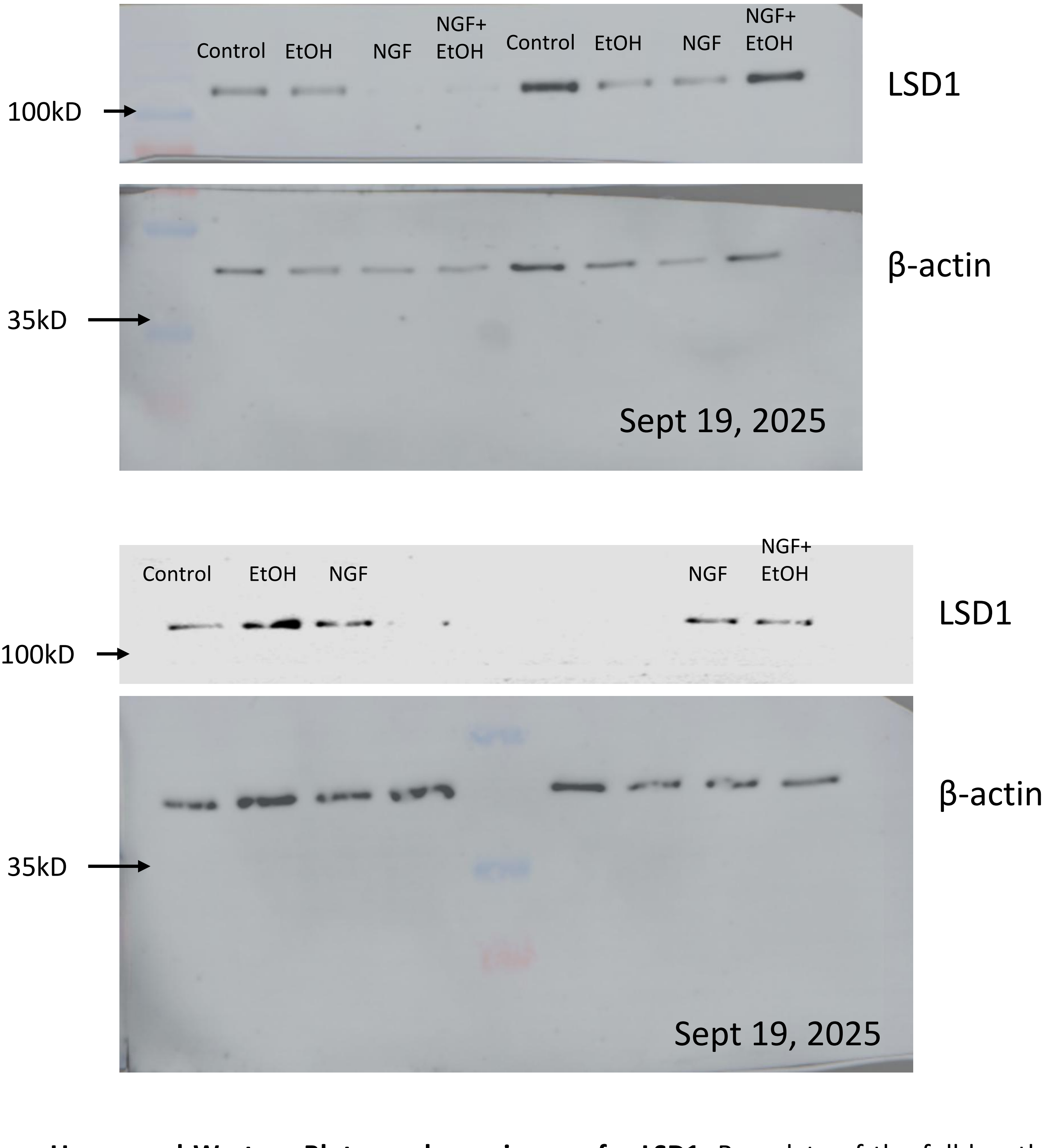

Uncropped Western Blot membrane images for LSD1. Raw data of the full-length membranes used for the quantification of LSD1 protein levels shown in Figure 2. PC12 cells were induced to undergo neuronal differentiation with NGF for 6 days and concurrently treated with ethanol during the final 5 days of the differentiation process (see experimental timeline in Fig. S1). Molecular weight markers and the specific bands corresponding to the analyzed isoform are indicated.
